# Increased Membrane Cholesterol in Lymphocytes Diverts T-Cells toward an Inflammatory Response

**DOI:** 10.1371/journal.pone.0038733

**Published:** 2012-06-19

**Authors:** Jacqueline Surls, Cristina Nazarov-Stoica, Margaret Kehl, Cara Olsen, Sofia Casares, Teodor-D. Brumeanu

**Affiliations:** 1 Department of Medicine, Division of Immunology and the Biostatistics Counseling Center, Uniformed Services University of the Health Sciences, Bethesda, Maryland, United States of America; 2 Infectious Diseases Directorate–Malaria Program, Naval Medical Research Center, Silver Spring, Maryland, United States of America; University of Oslo, Norway

## Abstract

Cell signaling for T-cell growth, differentiation, and apoptosis is initiated in the cholesterol-rich microdomains of the plasma membrane known as lipid rafts. Herein, we investigated whether enrichment of membrane cholesterol in lipid rafts affects antigen-specific CD4 T-helper cell functions. Enrichment of membrane cholesterol by 40–50% following squalene administration in mice was paralleled by an increased number of resting CD4 T helper cells in periphery. We also observed sensitization of the Th1 differentiation machinery through co-localization of IL-2Rα, IL-4Rα, and IL-12Rβ2 subunits with GM1 positive lipid rafts, and increased STAT-4 and STAT-5 phosphorylation following membrane cholesterol enrichment. Antigen stimulation or CD3/CD28 polyclonal stimulation of membrane cholesterol-enriched, resting CD4 T-cells followed a path of Th1 differentiation, which was more vigorous in the presence of increased IL-12 secretion by APCs enriched in membrane cholesterol. Enrichment of membrane cholesterol in antigen-specific, autoimmune Th1 cells fostered their organ-specific reactivity, as confirmed in an autoimmune mouse model for diabetes. However, membrane cholesterol enrichment in CD4^+^ *Foxp3* ^+^ T-reg cells did not alter their suppressogenic function. These findings revealed a differential regulatory effect of membrane cholesterol on the function of CD4 T-cell subsets. This first suggests that membrane cholesterol could be a new therapeutic target to modulate the immune functions, and second that increased membrane cholesterol in various physiopathological conditions may bias the immune system toward an inflammatory Th1 type response.

## Introduction

Plasma membrane cholesterol plays a critical role in cell signaling by stabilizing protein receptors within close proximity to liquid-ordered phase microdomains called lipid rafts [1]. With few exceptions, most functional receptors are sequestrated within lipid rafts prior to ligand ligation and downstream signaling [2]. Lipid rafts constitute about 30–40% of mammalian cell membranes [3]–[6]. Ligand-mediated clustering of neighboring receptor subunits into rafts leads to the assembly of fully functional receptors able to signal for T cell development, maturation, activation, and differentiation [7]–[10]. These processes occur upon formation of T cell-APC immunological synapse and TCR- peptide-MHC complex interactions [11], [12]. Clustering of rafts leading to T cell-APC immunological synapse formation may also occur in a non antigen-specific manner upon cross-linking of GM1 gangliosides by bacterial proteins such as cholera toxin B-subunit [13]–[15] or cross-linking of carbohydrate moieties of various protein receptors by galectins [16], [17].

Un-esterified cholesterol is the major component of lipid rafts, and its content is homeostatically regulated by extracellular uptake from blood circulating LDL [18], [19] and *de novo* intracellular synthesis [20]. Fine alterations in the amount of membrane cholesterol lead to re-organization of the lipid raft architecture, which in turn affects many T-cell functions such as proliferation [21], [22], blunting IL-2 production, or TCR signaling [23], [24] by incomplete or defective coupling of CD3/TCR receptor with its signaling modules [25]–[27]. Alterations in the cholesterol-rich lipid rafts of T-cell membranes can be induced *in vitro* either by depletion of cholesterol using cyclodextrins [28], or by cholesterol addition using hydroxypropyl-β-cyclodextrin [29]. Current therapeutic strategies aimed at immune regulation may also alter membrane cholesterol content, and thus inadvertently modify the architecture of protein receptors. Thus, glucocorticoids suppress the immune system and reduce inflammation in several disease settings, in part, by preventing compartmentalization of raft-associated proteins like LAT, LCK, FYN [30]. We and others show that certain therapeutic HMG-CoA reductase inhibitors like Lovastatin, a cholesterol lowering drug, can also lower the amount of T-cell membrane cholesterol and result in altered TCR signaling [31], [32]. Though some pharmaceutical immune regulatory agents lower membrane cholesterol while aiming to down-regulate of T-cell function, little is known about the effect of membrane cholesterol enrichment on T-cell function.

Early studies using *in vivo* administration of radio-labeled squalene demonstrated its ability to integrate into the cholesterol biosynthetic pathway and generate cholesterol [33]. Squalene is a late precursor of intracellular cholesterol synthesis that is being used as an adjuvant to enhance the human immune responses upon vaccination [34], but its immunomodulatory mechanism(s) remain largely unknown. Reports indicate that squalene treatment, or as little as 1% of dietary squalene, can limit the development of preneoplastic lesions involved in colon carcinogenesis [35–37]. In humans, squalene is ubiquitously found in the blood at very low concentrations due to its rapid turnover [38], while in the skin and adipose tissue at higher concentrations [39].

Herein, we questioned whether enrichment of membrane cholesterol in rafts by squalene administration affects cell differentiation of antigen-specific CD4 T-helper cells and suppressogenicity of CD4 *Foxp3* T-regulatory (T-reg) cells in mice expressing antigen-specific CD4 T-cells and CD4 *Foxp3* T-reg cells. A single dose of squalene, well below the liver toxicity level, was followed by a significant increase in membrane cholesterol by most lymphocyte subsets, particularly by the CD4 T helper and CD4 *Foxp3* T-regulatory (T-reg) cells. This increase in membrane cholesterol was paralleled by an increase in the number of resting CD4 T-cells in the spleen, and it favored Th1 differentiation of CD4 T-cells while having no effect on the suppressogenic function of CD4 *Foxp3* T-regulatory cells.

## Material and Methods

### Mice

To generate mice bearing antigen-specific CD4 T-cells and (GFP)-labeled CD4^+^ *Foxp3* ^+^ T-regulatory cells, the HA-specific TCR transgenic (TCR-HA Tg) mice expressing the 14.3d T-cell receptor that recognizes the HA_110–120_-CD4 T-cell immunodominant epitope of the hemagglutinin protein (HA) from the PR8/A/34 influenza virus [40] were crossed with BALB/c mice expressing GFP under the *Foxp3* promoter (Bar Harbor, Maine, USA). The F1 hybrids (*Foxp3*-GFP^+/−^, TCR-HA^+/−^) were used as the source of antigen-(HA) specific CD4 T helper and *Foxp3* ^+^CD4 T-reg cells. Since the *Foxp3* gene is expressed on X chromosome, *Foxp3*-GFP^+/+^ homozygous females were crossed with TCR-HA^+/+^ homozygous Tg males to obtain 100% heterozygous F1 offspring for both the TCR-HA and *Foxp3*-GFP transgenes. The F1 hybrids were genotyped by PCR and analyzed by FACS for *Foxp3*, GFP, and TCR-HA expression in CD4 T-cells. Some 30–35% of CD4^+^ T-cells and CD4^+^ *Foxp3*-GFP^+^ T-cells expressed TCR-HA in the spleen of F1 hybrids as measured by FACS using a TCR-HA_110–120_ clonotypic mAb (#6.5 mAb). The RAG2 KO, RIP-HA Tg mice expressing the PR8 influenza HA viral protein in the pancreatic β-cell islets under the rat insulin promoter [40] were used in adoptive cell transfer experiments as a read-out system for the HA-specific (diabetogenic) function of TCR-HA CD4 T-cells and for the regulatory function of CD4 *Foxp3* T-reg cells. Adoptively transferred TCR-HA Tg T-cells in RAG2 KO, RIP-HA Tg mice induces fulminate diabetes within two weeks post transfer [41], while the HA-specific CD4 *Foxp3* T-reg cells have a down-regulatory effect on diabetes onset [42], [43]. All mice were housed in pathogen-free conditions at Uniformed Services University of Health Sciences/Laboratory Animal Medicine facility. Experiments and care/welfare were in agreement with the Federal and Local regulations under an approved Institutional Animal Care and Use Committee protocol at Uniformed Services University of Health Sciences.

### Cell Isolation

Single-cell suspensions of CD4^+^ or CD25^+^ Treg-depleted cells were prepared by negative sorting from the spleen of untreated or squalene treated mice. The CD25^hi^ expression was found on more than 90% of TCR-HA^+/−^, *Foxp3*-GFP^+/−^ T-reg cells by FACS using triple staining with CD4, *Foxp3*, and CD25-dye conjugates (BD Biosciences, San Jose, CA, USA). Negative sorting of cells was preferred for lipid rafts studies, since positive sorting using specific antibodies cross-link plasma membrane receptors leads to clustering and re-organization of rafts, thereby introducing errors in data interpretation. The CD4^+^ splenocytes were negatively-sorted on mouse CD4 columns (R&D Systems, Minneapolis, MN, USA) according to the manufacturer’s instructions. To obtain CD4 T-cells devoid of CD4^+^ CD25^+^ (TCR-HA^+/−^, *Foxp3*-GFP^+/−^ T-reg) cells, negatively-sorted CD4 splenocytes were incubated at room temperature for 30 min. with CD25 Ab (5 µg/10^6^ cells, mAb clone# 7D4, ATTC) followed by incubation with a PE-labeled anti-CD25 Ab and enrichment on anti-PE antibody-magnetic beads according to the manufacturer’s instructions (Miltenyi Biotech, Auburn, CA, USA). The effluent (untouched) cell fraction was collected as the CD4^+^T-reg-depleted cell population. The *Foxp3*-GFP T-reg cells from the spleen of *Foxp3*-GFP^+/−^ TCR-HA^+/−^ (F1) hybrids were also negatively-sorted by FACS based on GFP fluorescence. In some experiments, the HA-specific T-cells [TIGHER]from both the TCR-HA Tg mice and *Foxp3*-GFP^+/−^ TCR-HA^+/−^ (F1) mice were FACS-sorted in cell preparations stained with the 6.5 mAb clonotypic mAb conjugated to an APC fluorochrome. The clonotypic 6.5 mAb is a rat IgG1 that recognizes the TCR-HA_110–120_ on T-cells. It does not cross-link the TCR-HA or the rafts, nor trigger TCR signaling [44]. To isolate splenic APCs, single-cell suspensions of splenocytes were incubated on a plastic surface in RPMI for 2 h at 37°C, 5% CO_2_. The adherent cells were detached by trypsinization, washed in PBS, and then rested in RPMI for 2 h before assayed.

### In Vivo Protocols

#### Squalene treatment

*Foxp3*-GFP^+/−^, TCR-HA^+/−^ double transgenic (dTg) mice (F1 hybrids) were injected intraperitoneally (i.p.) with one or four doses of 180 µg squalene (Sigma, Atlanta, GA, USA) emulsified 3∶1 in saline. At various time-points after the last injection, the blood, liver and spleen were collected for histology, *in vitro* bioassays, serum lipid electrophoresis, FACS analysis, and adoptive cell transfer experiments. Several splenic lymphocyte subsets were analyzed for the amount of membrane cholesterol by FACS at various time-points after the last injection.

#### Adoptive cell transfer

2×10^5^ or 10^4^ negatively-sorted CD25-depleted CD4^+^ splenic cells (diabetogenic T-cells), or representing or CD4 *Foxp3*-GFP^+/−^ T-cells (T-reg cells) from *Foxp3*-GFP^+/−^ TCR-HA^+/−^ (F1) mice were transferred or co-transferred i.p. into RAG2 KO RIP-HA Tg mice. The onset of autoimmune diabetes in RAG2 KO RIP-HA Tg recipients was monitored based on blood glucose values using Accu-Check glucose meter and glucose test strips (Roche, Indianapolis, IN, USA). Mice were considered diabetic after two consecutive readings of glycemia above 200 mg/dL.

### Western Blot Analyses

Single-cell suspension of splenocytes or negatively-sorted CD4^+^ splenocytes (8.5×10^7^) from untreated or squalene treated (180 µg/mouse) F1 mice were prepared 3 days post-squalene injection. Cells were washed with PBS and lysed for 30 min on ice in a buffer containing 0.1% SDS, 0.5% NP-40, 10 mM Tris, 150 mM NaCl, 1 mM Na_3_VO_4_, 16 mM EDTA, and a cocktail of protease inhibitors (pH 7, Roche, Indianapolis, IN, USA). Protein cell extracts were analyzed by SDS-PAGE and Western blot under denaturing/reducing conditions (1% SDS/0.5% 2-ME) using 8–25 gradient Phastgels (GE Life Sciences, Piscataway, NJ, USA) or 10–20% Tris-HCl Ready gels (BioRad, Hercules, CA, USA). Gels were either silver-stained or electrotransferred onto PVDF membranes (Millipore, Billerica, MA, USA). Transferred membranes were blocked with 5% BSA in PBS and probed with anti-mouse phosphotyrosine-HRP conjugate (R&D). Aliquots of protein cell extracts were precipitated either with anti-mouse CD3, CD28, IL-4Rα, IL-2Rα, or IL-12Rβ2 Abs for 2 h at room temperature, the immunoprecipitates were isolated by incubation with agarose-protein A/G beads (Pierce, Rockford, IL, USA) for 2 h at room temperature, and the beads were washed twice in PBS, and then boiled for 5 min in SDS-2ME sample buffer followed by a 5 min centrifugation at 5,000 rpm to remove the agarose beads. Supernatants were then electrophoresed, and electrotransferred onto PVDF membranes (Millipore, Auburn, CA, USA) blocked with 5% BSA in PBS and probed with pan-specific Tyr-phosphorylated HRP-labeled mouse IgG1 Ab (clone #179003, R&D), or with Abs specific for phosphorylated ZAP-70, PI3K, STAT-6, STAT-4, or STAT-5 (Abcam, Cambridge, MA, USA). PVDF membranes were washed, and bound anti-phospho Abs were detected by isotype specific secondary anti-IgG-HRP Ab-conjugates (Cell Signaling). The HRP signal was visualized in a Kodak Imaging Analyzer by chemiluminescence using a Pierce ECL western blotting substrate (Pierce, Rockford, IL, USA) according to the manufacturer’s instructions.

### T Cell Bioassays

Single-cell suspensions of splenocytes (10^6^ cells) from individual mice were incubated in round bottom 96-well plates with RPMI complete media containing HA_110–120_ synthetic peptide (40 µg/mL) at 5% CO_2_ and 37°C. Cytokine secretion in the cell culture supernatants was measured by Luminex, i.e., 24 h of stimulation for IL-2 secretion and 36 h of stimulation to measure IL-4 and IFN-γ secretion. For the APC-CD4 T-cell co-culturing experiments, the CD4 T cells (10^6^) and APCs (5×10^5^) from untreated or squalene treated mice were pulsed or not with HA_110–120_ synthetic peptide (40 µg/mL). For the T-cell suppression experiments, T-regs were co-cultured in 96-well plates with HA-specific CD4 T-cells at 1∶1 ratio (2×10^6^ total cell number/well) in the presence of HA_110–120_ peptide (40 µg/mL) for 48 h at 5% CO_2_ and 37°C, and the IL-4 and IFN-γ secretion in cell culture supernatants was measured by Luminex. Cytokines concentration was measured in Multiplex mouse cytokines kits using a Luminex instrument (Luminex Corporation, Austin, TX, USA) and a 5 parameter logistics model equation (MasterplexQT software, Miraibio, San Francisco, CA, USA) according to the manufacturer’s instructions.

### Histology

The liver or spleen from untreated or squalene treated mice was frozen in Tissue-Tek O.C.T. compound (VWR, Batavia, IL, USA). Frozen liver sections were mounted on glass slides, permeabilized with digitonin, and stained for cholesterol with hematoxylin-eosin and Sudan IV (Histoserv, Germantown, MD, USA). Images were captured using the Eclipse Nikon microscope and NIS-Elements AR Nikon software (Nikon, Melville, NY). To identify cytosolic cholesterol accumulation and lipoidic microdroplets, frozen spleen sections were mounted on glass slides and stained with Oil-red O staining (ORO) and hematoxylin-eosin. Briefly, mounted spleen sections were stained in 36% Oil red O/Triethyl phosphate working solution for 10 min (Sigma-Aldrich, Atlanta, GA, USA), washed in tap water, and then counterstained with Harris’ hematoxylin (Sigma-Aldrich, Atlanta, GA, USA). Slides were mounted with VectaShield Hard Set Mounting medium containing DAPI (Vector Laboratories, Burlingame, CA, USA) and analyzed by phase contrast microscopy using a Zeiss instrument (Thornwood, NY, USA).

### Phase Contrast and Confocal Laser Scanning Microscopy (CLSM)

Negatively-sorted CD4 T-cells (10^6^ cells) from the spleen of individual mice previously treated or not with squalene were co-stained with 1.5 µl CTB-FITC conjugate (Sigma) and anti-CD4 Ab-PE conjugate (BD Biosciences) for 30 min at 4°C, on ice. CTB is a specific ligand for the GM1 glycosphingolipid resident moieties of the lipid rafts [45]. To visualize the IL-2Rα, IL-4Rα, or IL-12Rα subunits that co-localize with the lipid rafts of negatively-sorted CD4 T-cells (10^6^ cells), single cell suspensions (10^6^ cells) were co-stained with specific anti-receptor Ab-PE conjugates (BD Biosciences) and CTB-FITC conjugate. Stained cells were washed twice in PBS/BSA 1% and mounted onto glass slides using Vectashield containing DAPI stain (Vector Laboratories Inc., Birmingham, CA) to identify cell nuclei. Distribution of GM1 resident moiety in the cholesterol-rich rafts of plasma membrane was captured as 2D images, and the GM1 content was measured based on CTB-FITC cell intensity using a ZEISS 710 Confocal Laser Scanning Microscope equipped with ZEISS ZEN 2009 analysis software (Thornwood, NY, USA). To analyze receptor co-localization with the lipid rafts, resting CD4 T-cells from the squalene treated or untreated mice were triple stained with DAPI, GM1-CTB, and either anti IL-2Rα-APC, anti-IL-4Rα-PE, or anti-IL-12Rβ2-PE conjugates (BD Biosciences).

### Flow Cytometry

Single-cell suspension of splenocytes (10^6^ cells) from untreated or squalene treated mice were stained for 30 min at 4°C for surface markers or intracellular cytokine synthesis using specific antibody-dye conjugates (BD Biosciences) or their isotype controls. In some experiments, cells were co-stained with Filipin III (Sigma) to measure the amount of cholesterol in the plasma membrane. Mean fluorescence intensity (MFI) of Filipin III or Ab-dye conjugates was measured by FACS at the single-cell level in 10^4^–10^5^ cell events acquired with a LSR II Becton-Dickinson instrument equipped with the WINLIST analysis software (Verity, Topsham, ME, USA). FACS measurements of membrane cholesterol was estimated by the MFI values of Filipin III as described [46]. Filipin III binds specifically to un-esterified cholesterol present in the plasma membrane of lipid rafts [47]. Spectrophotometric measurements between λ_excitation_ = 340 and 380 nm and λ_emission_ = 385–470 nm ruled out the possibility that Filipin may stain squalene possibly attached or incorporated into the plasma membrane.

### Real-time RT-PCR

Total RNA and cDNA from splenocytes was extracted using Pure-Link Micro and Midi RNA Purification Systems (Invitrogen, Carlsbad, CA, USA) and High Capacity cDNA Archive kit (Applied Biosystems, Foster City, CA, USA), respectively. The primers for murine HMG-CoA reductase were forward-5′GAATGCCTTGTGATTGGAGTTG3′, and reverse- 5′GCCGAA GCAGCACATGATCT3′; specific primers for Squalene epoxidase, T-bet, GATA-3, IL-2Rα, IL-12Rβ2, and IL-4Rα were purchased from Applied Biosystems. Measurement of gene products expression was carried in an ABI Prism 7700 equipped with SDS 1.9.1 analysis software (Applied Biosystems), as previously described [48]. The relative mRNA levels were estimated in reference to the 18S rRNA (Applied Biosystems).

### Serum Lipid Electrophoresis

Fresh mouse serum from untreated or squalene treated mice was assessed for the fractions of high density lipoproteins (HDL), low density lipoproteins (LDL), and very low density lipoproteins (VLDL) using the QuickGel Cholesterol kit and an electrophoretic system from Helena Laboratories (Beaumont, TX, USA) according to the manufacturer’s protocol. The lipoprotein fractions were quantified based on the number and intensity of pixels per each electrophoretic band using the SCION Image analysis software (Scion Corp. Frederick, MD, USA).

### Statistics

Significance of results between untreated and squalene treated groups for the MFI values measured by FACS, cytokines concentration in Luminex, RNA expression level in RT-PCR, and blood glucose values, was determined by the unpaired Student’s *t*-test for which *p values less than 0.05 were considered significant. For each cytokine, means were also compared across groups using one-way analysis of variance (ANOVA) followed by Tukey’s post-hoc pairwise comparisons. *p values<0.05 were considered statistically significant.

## Results

### Squalene Treatment Up-regulates Membrane Cholesterol in Various Lymphocyte Subsets

Squalene is a late cholesterol precursor that can be efficiently taken up by hepatocytes and converted into cholesterol when administered intravenously [33]. Herein, we measured the content and distribution of membrane cholesterol in various splenic lymphocyte subsets following i.p. injection of squalene (emulsified 3∶1 in saline) in F1 hybrid mice (TCR-HA^+/−^, *Foxp3*-GFP^+/−^ mice).

**Figure 1:**
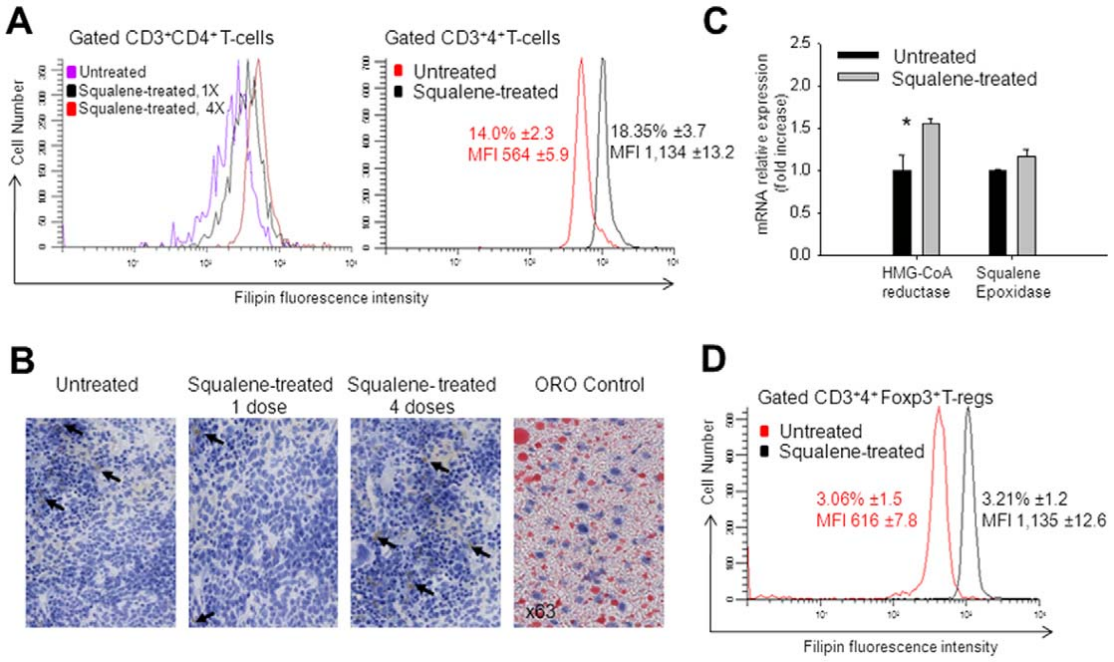
Squalene administration leads to accumulation of membrane cholesterol in resting CD4 T-cells. (**A**) F1 hybrid mice (n = 5/group) were injected i.p. or not (purple line) with a single dose (black line) or 4 doses of squalene (red line) within a week interval (180 µg/dose/mouse). Seven days after the last injection, negatively-sorted splenic CD4 cells from individual mice were co-stained with CD3-PE, CD4-FITC and Filipin III. Shown is the amount of cholesterol in plasma cell membrane of gated CD3^+^CD4^+^ splenic cells as measured by MFI of Filipin III in FACS at single-cell level in one representative mouse from each group (*left panel*). *Right panel*, F1 hybrid mice (n = 7/group) were injected i.p. (black line) or not (red line) with a single dose of squalene (180 µg/mouse) and 7 days later negatively-sorted splenic CD4 cells from individual mice were co-stained with CD3-PE, CD4-FITC, and Filipin III. Shown is the percentage of gated CD4^+^ T-cells ± standard deviation (SD) and MFI values of Filipin III ± SD before and after squalene injection as collected among 700 cell events in gated population of CD3^+^4^+^ T-cells for one of three representative experiments. (**B**) Cholesterol accumulation in the spleen was identified by Oil Red O (ORO) staining of frozen spleen sections, counter-stained with hematoxylin from untreated or squalene treated mice (180 µg/mouse) given one or four doses, and analyzed 7 days post-injection (n = 3/group). *Left panel,* spleen section from untreated mouse. *Middle panels,* spleen section from squalene treated mice. *Right panel,* positive control for ORO lipid droplet staining in adipocytes. Shown is one representative ORO stained section in each group. Dark arrows indicate ORO stain. (**C**) Quantitative real-time RT-PCR of HMG-CoA reductase mRNA and Squalene epoxidase mRNA extracted from negatively-sorted CD4 splenocytes isolated from individual F1 hybrid mice (n = 5/group) that were treated (light bars) or not treated (dark bars) with a single dose of squalene (180 µg/mouse) and analyzed 7 days post-injection. Y axis indicates the mean fold increase in mRNA expression level relative to the endogenous 18S rRNA expression level (control ± standard deviation (SD). Shown are two combined separate experiments (*p value<0.05). (**D**) FACS measurements of CD3^+^4^+^ *Foxp3* ^+^ T-reg cells from negatively-sorted CD4^+^ splenic cells of the same F1 mouse groups analyzed in panel A that were co-stained with CD3-PE and Filipin III. Shown is the percentage of gated CD4^+^Foxp3^+^ T-reg cells ± SD and MFI values of Filipin III ± SD collected among 500 cell events in the gated population of GFP^+^-*Foxp3*/GFP^+^ cells from one mouse in each group from two representative experiments.

F1 mice were treated i.p. with 0, 1, or 4 doses of squalene (180 µg/dose). Seven days after the last injection, negatively-sorted, resting CD4 splenic T-cells from each group were stained with filipin and analyzed for the amount of membrane cholesterol by FACS. The kinetics of membrane cholesterol accumulation in resting CD4 T-cells from F1 mice given a single dose of squalene (180 µg/mouse) indicated that the peak cholesterol load occurs after 7 days, with a gradual decrease within the following two weeks till reaching physiological levels (data not shown). Four injections of squalene (180 µg/dose/mouse) administered within a week interval significantly increased the amount of membrane cholesterol in CD4 T-cells (45% MFI increase) as compared with those from untreated (control) mice. However, no significant difference was observed between mice given one or four doses of squalene (40% *vs.* 45% MFI increase) (**Figure 1A**). This may be explained by the saturation limit of either the cellular uptake of squalene or the intracellular trafficking of cholesterol from the cytosol to the plasma membrane in CD4 T-cells. A slight homogenous accumulation of cholesterol in the spleen was visualized by phase contrast microscopy in oil-red-o stained cross-sections from F1 mice treated with 4 doses but not a single dose of squalene (**Figure 1B**). Membrane cholesterol accumulation in resting CD4 T-cells upon squalene administration appeared to be an active process regulated by genes involved in cholesterol synthesis, since an increase in the mRNA expression levels of HMG-CoA reductase and Squalene epoxidase by 30% and 15% respectively, was detected 3 days after a single dose of squalene (**Figure 1C**).

We have previously shown that accumulation of membrane cholesterol in resting CD4 T-cells is a developmental feature and that the content of membrane cholesterol is stable between 2 and 4 months of age in mice [49]. To avoid physiological alterations in T-cell membrane cholesterol content, all experiments were carried out in 3 month-old F1 mice when the amount of membrane cholesterol is most stable.

Seven days after a single dose of squalene (180 µg/mouse), single-cell level flow cytometry analysis revealed that the amount of membrane cholesterol in CD4 *Foxp3* splenic T regulatory cells was also increased by 40 to 50% (**Figure 1D**). Resting B-cells, and dendritic cells from the spleen of F1 mice given a single dose of squalene (180 µg/mouse) also showed an increase (5–10%) in membrane cholesterol, albeit less dramatic when compared to CD4 and CD8 T-cells (**Figure S1**).

**Figure 2:**
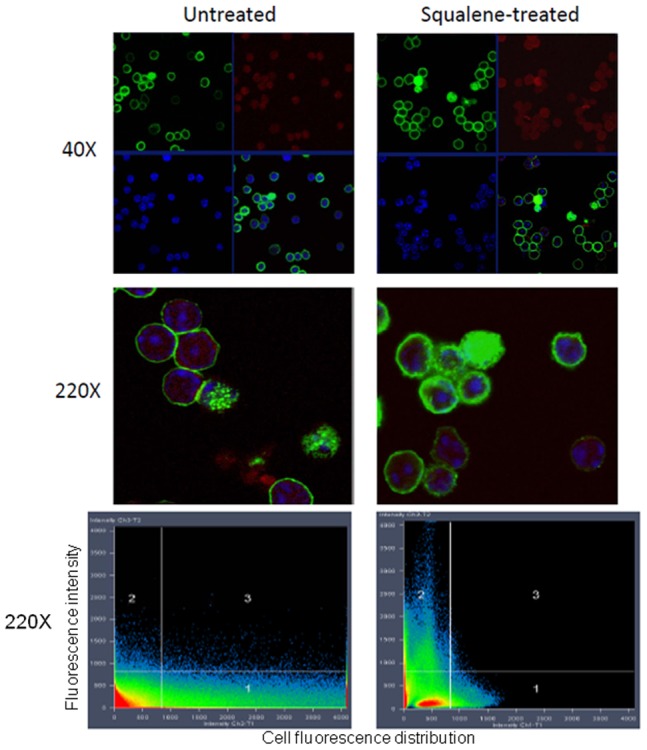
Squalene induced accumulation of membrane cholesterol in resting CD4 T-cells alters the partitioning of lipid rafts. Negatively-sorted CD4^+^ splenocytes were isolated from the spleen of untreated or squalene treated (single 180 µg dose/mouse) F1 mice 7 days post-injection. Cells were co-stained with CD4-PE, CTB-FITC and DAPI and analyzed by CLSM. *Upper panels,* quadrants indicating single-color stained cells at X40 magnification from untreated (*left column*) and squalene treated F1 mouse (*right column*): *upper left* quadrant, CTB-FITC staining (green); *upper right* quadrant, CD4-PE stain (red); *lower left* quadrant, DAPI staining of nuclei (blue), and *lower right* quadrant, merged channels for CTB-FITC, CD4-PE and DAPI staining. *Middle panels*, merged CTB-FITC, CD4-PE and DAPI staining of clusters of splenocytes from untreated and squalene treated F1 mouse at X220 magnification. *Bottom panels,* color intensity quantification for cell clusters shown in the middle panels using the using the ZEISS ZEN 2009 analysis software. Of note, the amount of GM1 moiety revealed by CTB-FITC (green) in merged channel 2 is significantly higher for the CD4 T-cells from squalene treated mouse than that of the untreated mouse. Shown is one of three mice analyzed in each group.

A single-dose of squalene administration (180 µg/mouse) was consistently associated with altered CD4 T-cell frequencies within the pool of resting splenocytes, as analyzed by flow cytometry 7 days post-injection. Thus, the number of resting CD4 T-cells was increased by 20–22%, whereas the number of CD4 *Foxp3* T-cells, CD8 T-cells, CD19^+^ B-cells, and CD11c^+^ dendritic cells remained relatively unchanged (**Figures 1A** (right panel), **1D and S1**). Furthermore, CLSM analysis of the resident lipid raft marker GM1 in negatively-sorted, resting CD4 splenic cells from F1 mice injected with a single dose of squalene (180 µg/mouse) showed that membrane cholesterol enrichment was associated not only with a higher GM1 expression, but also with a more homogeneous distribution as compared with lower GM1 expression and more heterogeneous distribution in resting CD4 splenic cells from untreated mice (**Figure 2**). A narrow fluorescence intensity peak observed by CLSM analysis (**Figure 2**, bottom right panel) indicates a narrow range of GM1 homogeneity within a given cell population, such that the majority of cells express the same amount of GM1. In contrast, a broad fluorescence intensity peak refers to a wide range of GM1 heterogeneity within a given cell population. Thus, the cell population is comprised of a mixture of cells expressing high, intermediate, low, and very low amount of GM1. (**Figure 2**, bottom left panel).

To determine the extent to which *in vivo* squalene administration may affect cholesterol metabolism in the liver, the electrophoretic profile of serum cholesterol fractions and rate of cholesterol accumulation in liver were analyzed. F1 mice given a single dose of squalene (180 µg/mouse) showed no significant alterations in the ratio of serum cholesterol fractions or cholesterol accumulation in the liver 7 days post-injection (data not shown). However, four doses of squalene (180 µg/dose/mouse) administered once a week led to an increase in the high density lipid fraction (HDL) in serum 7 days after the last injection (**Figure S2-A**), and a modest accumulation of cholesterol in liver tissue (**Figure S2-B**). Of note, all biofunctional assays in this study were carried out in 3 month-old F1 mice injected with a single i.p. dose of squalene (180 µg/mouse), for which no alterations in serum cholesterol fractions or liver cholesterol accumulation were detected. Early studies showed that LDL serum cholesterol increases upon inhibition of hepatic squalene synthesis by zaragozic acid [50]. In contrast, we found that squalene administration was paralleled by an increase in HDL serum cholesterol.

In summary, these results indicate that exogenous squalene can be efficiently taken up by various subsets of resting peripheral lymphocytes and is constantly associated with an increase in membrane cholesterol, particularly in resting CD4 T-cells. Squalene administration was also followed by an increased frequency of resting CD4 T-cells (∼20% increase), but had no significant effect on the frequency of CD4 *Foxp3* T-reg cells, CD8 T-cells, B-cells, and dendritic cells in the spleen.

### Cholesterol Enrichment in CD4 T-cell Membrane Sensitizes the Th1 Signaling Modules in the Absence of T-cell Stimulation

**Figure 3:**
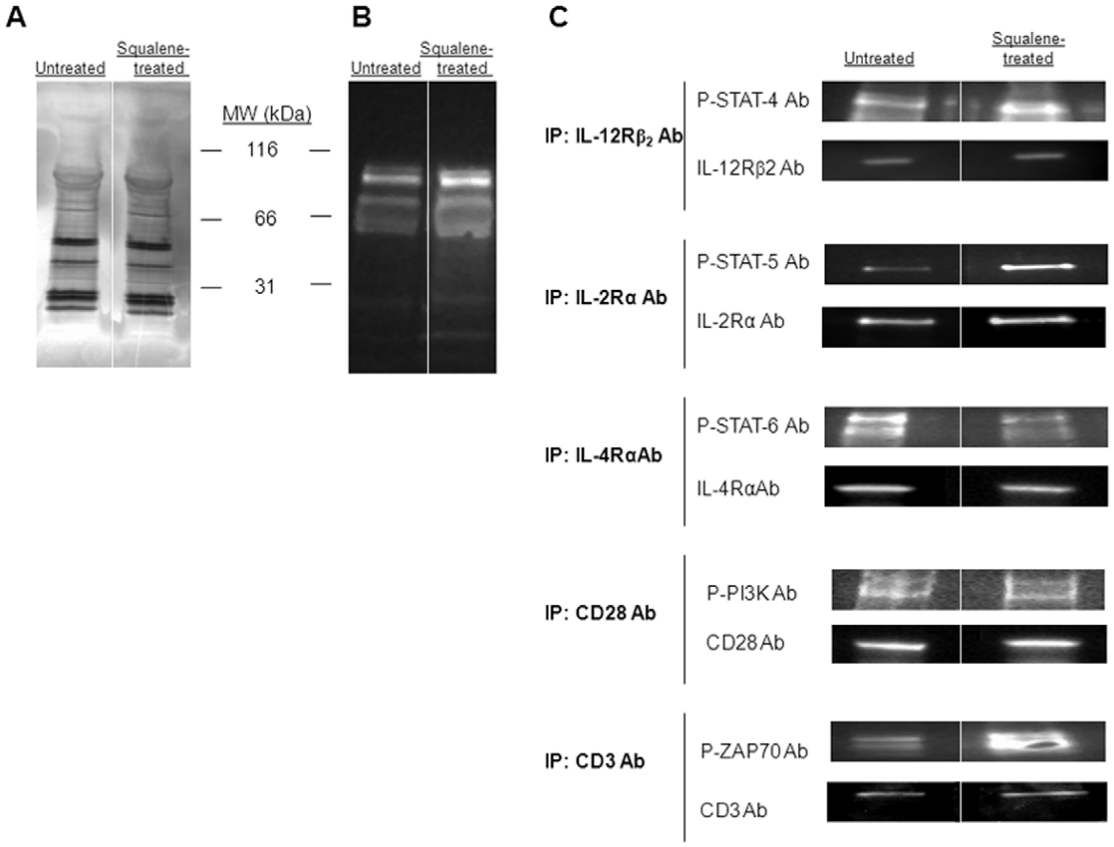
Tyrosine phosphorylation patterns of major signaling modules of T helper differentiation upon enrichment of membrane cholesterol. (**A**) SDS-PAGE silver stain of pooled, negatively-sorted CD4^+^ splenic T-cell lysates from F1 mice (n = 4/group) untreated (*left lane*) or squalene treated (single dose of 180 µg, *right lane*) 3 days post-injection shows no detectable quantitative alterations in the protein bands between the two groups of mice. (**B**) Tyrosine phosphorylation patterns of the same samples in panel *A* were blotted with anti-phosphorylated tyrosine Ab-HRP conjugate. Of note, the amount of 55–100 kDa phosphorylated protein bands is increased in mice treated with squalene. (**C**) Immunoprecipitation of pooled splenic CD4^+^ T-cell lysates from F1 mice treated or not with squalene (180 µg/mouse, n = 4/group) 3 days post-injection was carried out for IL-12Rβ2, IL-2Rα, IL-4Rα, CD28, or CD3 receptors using specific Abs, and probed with specific anti-phospho Abs for STAT4, STAT5, STAT6, PI3K, and ZAP-70 kinases. Only the phosphorylated STAT-4, STAT-5, and ZAP-70 in squalene treated mice were significantly enhanced. Shown is one of two representative experiments.

Cholesterol-rich plasma membrane microdomains (lipid rafts) play a critical role in early intracellular signaling towards many cell functions. These signaling events can be detected by phosphorylation of various protein receptors and their adaptor molecules, as well as by the recruitment of various kinases to their intracellular domains. Herein, we questioned whether enrichment of membrane cholesterol in resting CD4 splenic T-cells may alter the baseline of tyrosine phosphorylation in several signal transduction molecules critical for cell differentiation. First, the protein patterns from total lysates of negatively-sorted, resting CD4 splenic T-cells from untreated or squalene treated (180 µg/mouse) F1 mice showed no detectable quantitative alterations 3 days post-injection, as the number and intensity of SDS-PAGE protein bands were similar in both groups of mice (**Figure 3A**). However, a modest increase in the 55 to 100 kDa tyrosine phosphorylated proteins was detected in the T-cell lysates from squalene treated mice (**Figure 3B**).

Analysis of several protein kinases and transducer molecules required for downstream signaling of T-cell differentiation, i.e., IL-12R, IL-4R and their signaling molecules, revealed a differential modulatory effect of membrane cholesterol in resting CD4 T-cells in the absence of antigen stimulation (**Figure 3C**). During T-cell differentiation, binding of IL-12 to IL-12Rβ2 leads to STAT-4 phosphorylation in Th1 cells [51], whereas binding of IL-4 to IL-4R leads to STAT-6 phosphorylation in Th2 cells [52]. Data depicted in **Figures 3C and 3D** showed that resting CD4 splenic T-cells enriched in membrane cholesterol had an increased baseline of STAT-4 phosphorylation and a decreased baseline of STAT-6 phosphorylation. A number of additional signaling events supporting the T-cell growth and survival also occur during Th1 cell differentiation. Thus, binding of IL-2 to IL-2R promotes recruitment and phosphorylation of JAK kinases on the IL-2Rβ chain, which in turn mediates the assembly of a fully functional IL-2Rαβγ followed by STAT-5 phosphorylation, heterodimerization, and translocation to the nucleus where it binds to restriction elements in the gene promoter of several genes. [53], [54]. The basal level of phosphorylated STAT-5 was increased in resting CD4 T-cells upon enrichment of membrane cholesterol (**Figure 3C**). Furthermore, during Th1 differentiation the CD3 chains are assembled together with the T cell receptor (TCR) α and β chains in the cell membrane to form a fully functional CD3/TCR complex. The ζ-chain of CD3/TCR complex possesses phosphorylated ITAM motifs that are crucial for recruitment and docking of phosphorylated ZAP-70 [55]. Membrane cholesterol enrichment in resting CD4 splenic T-cells led to an increased base level of phosphorylated ZAP-70 kinase (**Figure 3C**). In addition to CD3/TCR and cytokine receptor signaling, CD28 co-stimulation through ligation by B7 molecules expressed by APCs provides sustained CD3/TCR signaling and promotes Th1 cell survival [55], [56]. An early event of CD28 ligation is the recruitment and phosphorylation of PI3K on its intra-cytoplasmic tail. Our data consistently showed that enrichment of membrane cholesterol in resting CD4 splenic T-cells had little if any effect on the phosphorylation and recruitment of PI3K to the CD28 intra-cytoplasmic tail (**Figure 3C**).

**Figure 4:**
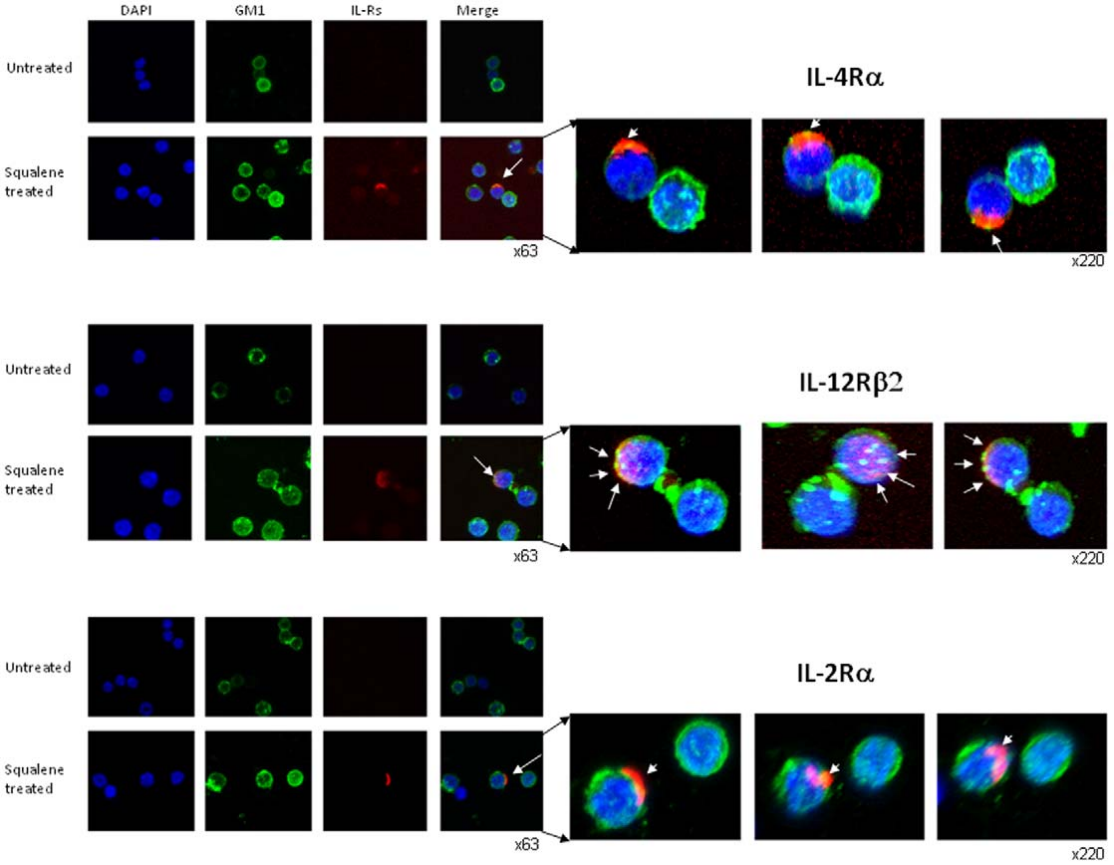
Co-localization of cytokine receptors with lipid rafts of resting CD4 T-cells before and after squalene treatment. Resting CD4 T-cells from individual F1 mice (n = 5/group) before and 7 days after squalene treatment (180 µg/mouse) were analyzed for interleukin receptor expression and distribution at the single-cell level by CLSM. Cells were stained with IL-4Rα-, IL-12Rβ2-, or IL-2Rα-PE conjugates, and co-stained for GM1 ganglioside by CTB- FITC conjugate and for nuclei with DAPI. First column indicates single-channel color for DAPI staining (blue), second column indicates GM1 staining (green), third column indicates interleukin receptor (IL-Rs) staining (red), and last column indicates merged channels at X63 magnification. *Top-two rows*, indicate cells from untreated (*upper row*) and squalene treated mice (*lower row*) stained for IL-4Rα. *Middle-two rows*, indicate cells from untreated (*upper row*) and squalene treated mice (*lower row*) stained for IL-12Rβ2. *Bottom-two rows*, indicate cells from untreated (*upper row*) and squalene treated mice (*lower row*) stained for IL-2Rα. Arrows indicate presence of IL-Receptor co-expression with the GM1 resident of lipid rafts. Enlargements of the merged channels are depicted to the *right* along with two different angles of the membrane for each IL-Receptor at X220 magnification. Shown are representative images in one of three experiments.

**Figure 5:**
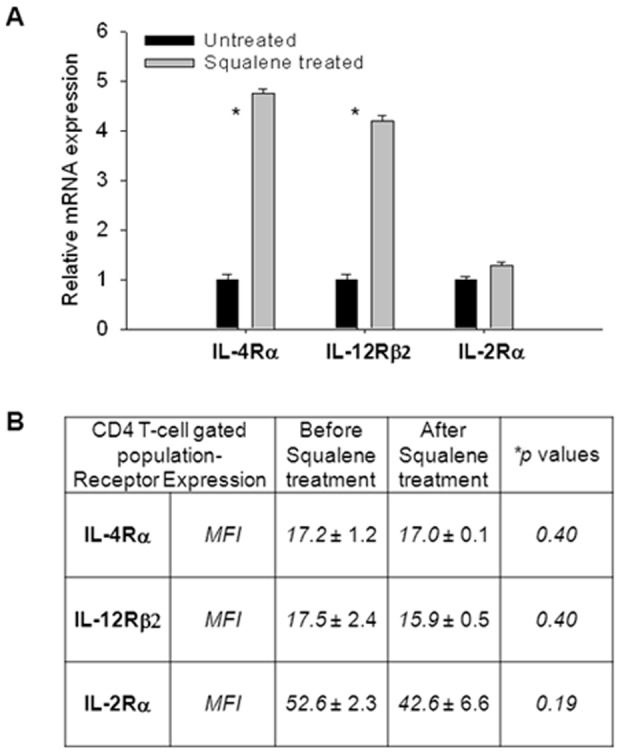
Alteration in cytokine receptors mRNA expression after squalene enrichment of membrane cholesterol in resting lymphocytes. (**A**) Quantitative real-time RT-PCR of IL-4Rα, IL-12Rβ2, and IL-2Rα mRNA extracted from peripheral blood lymphocytes of individual F1 mice (n = 5/group) analyzed before squalene treatment (dark bars) and 7 days after squalene injection (180 µg/mouse) (light bars). Y axis indicates the mean fold increase in mRNA expression level relative to the endogenous 18S rRNA expression level (control ± SD). Shown are two combined separate experiments (*p values<0.05). (**B**) Aliquots samples in panel A were stained with CD4-FITC conjugate, co-stained either with IL-4Rα-PE or IL-12Rβ2-PE or IL-2Rα-PE conjugates, and analyzed by FACS at the single-cell level for the surface IL-Rs expression level based on MFI measurements. Shown are the IL-Rs MFI values ± SD measured in individual mice before and after squalene treatment. Of note, no significant changes occurred in the IL-Rs expression on cell surface after squalene treatment (*p values>0.05).

Further analysis of resting CD4 splenic T-cells from F1 mice treated or not with squalene was carried out by CLSM to visualize the membrane distribution of IL-12Rβ2 and IL-4Rα subunits that recruit STAT-4 in Th1 differentiating cells and respectively STAT-6 in Th2 differentiating cells. CLSM analysis of IL-2Rα revealed a differential co-localization of this receptor subunit with the lipid rafts, as well as IL-4Rα and IL-12Rβ2, 7 days after squalene treatment. In differentiating CD4 T-cells, the autocrine IL-2 secretion and IL-2R up-regulation on cell surface play a critical role for the cell growth and proliferation. The first observation was that these receptor subunits were easily detected in the rafts upon squalene treatment (**Figure 4**). Secondly, the IL-2Rα and IL-4Rα subunits were observed as capping in rafts microdomains, whereas the IL-12Rβ2 subunit was more dispersed in the rafts (**Figure 4** **,** *enlargements*). Third, squalene treatment was paralleled by increased mRNA expression level, particularly for IL-4Rα and IL-12Rβ2 subunits in resting CD4 T-cells (**Figure 5A**). Fourth, the level of cell surface expression of either receptor was not significantly increased by squalene treatment as determined by the MFI values in single-cell level FACS analysis (**Figure 5B**). Conceivably, a differential receptor re-distribution in the rafts detectable by CLSM implies that the unique structure of each receptor and the amount of cholesterol in the microdomains could both play important roles. The mechanism by which spatiotemporal re-organization of IL-2R, IL-4R, and IL-12R in the rafts microdomains of resting CD4 T-cells upon membrane cholesterol enrichment remains to be further investigated.

Together, these data indicate that enrichment of membrane cholesterol in resting CD4 T-cells by 40 to 50% can sensitize the Th1 signaling machinery by means of rafts re-distribution of cytokine receptors and increased phosphorylation of signaling modules critical for Th1 cell differentiation.

### Cholesterol Enrichment in the CD4 T-cell Membrane Fosters Type 1 Cell Differentiation in the Presence of T-cell Stimulation

Since enrichment of membrane cholesterol in resting CD4 T-cells increased the phosphorylation of signaling modules involved in Th1 differentiation in the absence of T-cell stimulation, we next investigated whether enrichment of membrane cholesterol in CD4 T-cells and/or antigen presenting cells (APCs) under stimulation may affect the outcome of T-cell differentiation. For this, the cytokine secretion profiles from various combinations of negatively-sorted CD4 T-cells and APCs isolated from untreated or squalene treated (180 µg) F1 mice (HA_110–120_-specific TCR) were analyzed in the presence of antigen-specific (HA_110–120_) stimulation or non antigen-specific CD3/CD28 polyclonal stimulation.

**Figure 6:**
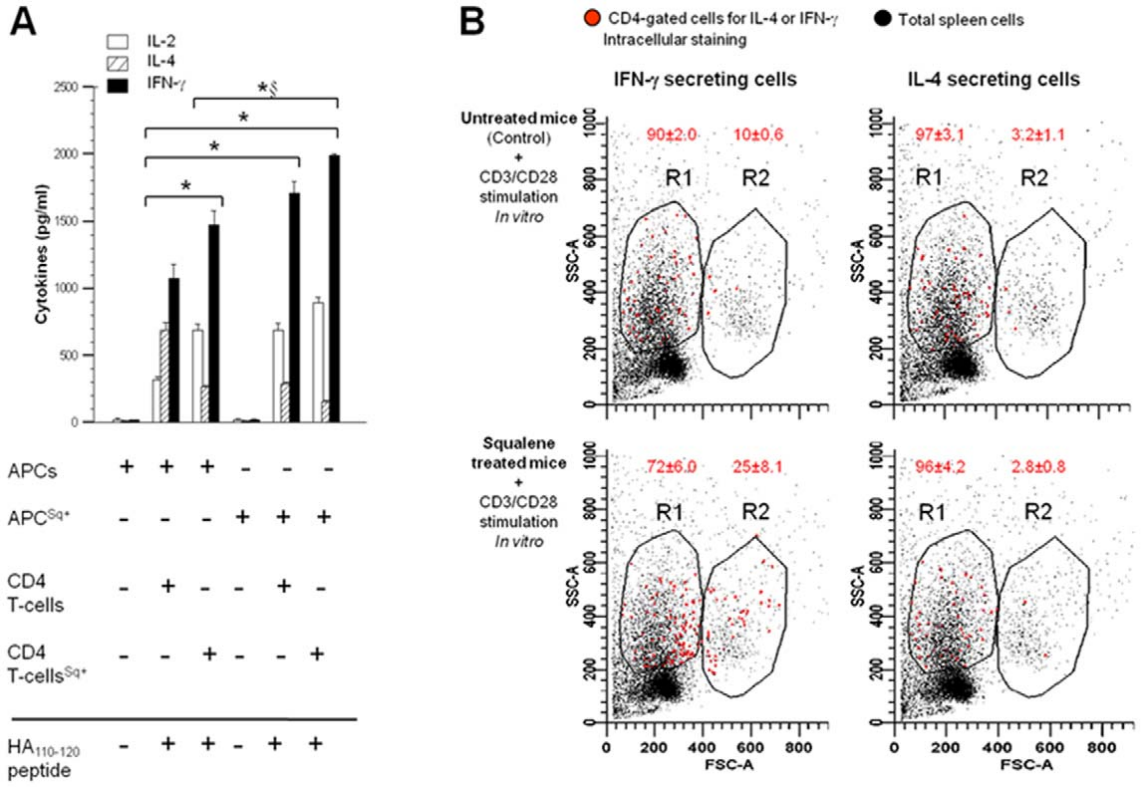
Squalene induced accumulation of membrane cholesterol in resting CD4 T cells favors Th1 polarization under antigen-specific or non antigen specific stimulation. (A) Isolated adherent splenocytes (APCs, 5×10^5^) from individual F1 mice treated or not with 180 µg of squalene, were pulsed (+) or not pulsed (-) with HA_110–120_ synthetic peptide (40 µg/mL/10^6^ cells) *in vitro* 7 days after squalene injection, and co-cultured with negatively-sorted CD4 splenic T-cells (10^6^ cells) from the same groups of mice, treated or not with squalene (Sq) (n = 4/group). Various cell co-culture combinations are shown on the X-axis, where (+) indicates presence and (–) indicates absence from the culture. Cell-culture supernatants were collected 24–48 h later, and secretion of IL-2, IL-4, and IFN-γ (Y-axis) was measured in pg/ml by Luminex. Bars represent average ± SD. Differences among groups were highly significant (*p*<0.001) for cytokines with the following exceptions denoted as §: For IL-4, co-culutre in lane 6 differed significantly from lane 5 (*p* = 0.047) but not from lane 3 (*p* = 0.097). No significant difference was observed between co-culture in lane 3 and 5 for any of the three cytokines. (**B**) Intracellular cytokine staining for IFN-γ (*left panels*) and IL-4 (*right panels*) in splenic cells and CD4-gated splenic cells from individual untreated (*top panels*) and squalene treated F1 mice (*bottom panels*) (n = 3/group) were stimulated for 48 h with anti-CD3/CD28 Abs (2.5 µg each/10^6^ cells). Shown are the overlapped FACS histograms of gated CD4^+^ T cells synthesizing IL-4 or IFN-γ (red cell events), and total splenic cells (dark cell events) from squalene treated or untreated F1 mice, 7 days after squalene treatment. R1 gate indicates the low-proliferating cell population whereas the R2 gate indicates the high proliferating cell population in each experiment. Dead cells are shown in the un-gated cell population below the R1 gate. Shown is one of two representative experiments.

Co-cultures of membrane cholesterol-enriched, resting CD4 T-cells and HA_110–120_ pulsed APCs enriched in membrane cholesterol showed the highest secretion of IL-2 and IFN-γ and the lowest IL-4 secretion (**Figure 6A**). The amount of IL-2 and IFN-γ secreted by resting CD4 T-cells stimulated with HA_110–120_-pulsed APCs was 30–35% lower when T-cells were not enriched in membrane cholesterol, indicating that membrane cholesterol enrichment in both CD4 T-cells and APCs provides the strongest Th1 polarization. Thus, while the HA-specific CD4 T-cells stimulated with HA_110–120_ peptide usually developed a Th1/Th2 mixed response, those from squalene treated mice developed a predominant Th1 response. Because membrane cholesterol enrichment in APCs augmented IFN-γ secretion by antigen-specific CD4 T-cells, and IL-12 secretion by APCs is known to up-regulate IFN-γ secretion by CD4 T-cells, we next compared the steady-state of IL-12 secretion by unstimulated APCs from untreated and squalene treated F1 mice. The baseline of IL-12 secretion was near the limit of detection in APCs cultured from untreated (control) mice, while being significantly higher in APCs cultured from squalene treated mice (**Figure S3**). This may well explain why IFN-γ secretion by CD4 T-cells was significantly augmented in the presence of HA-pulsed APCs enriched in membrane cholesterol. The base level of IL-6 secretion, but not IL-1α secretion by APCs was also slightly increased upon membrane cholesterol enrichment (**Figure S3**), which indicated a differential effect of membrane cholesterol not only on CD4 T-cell function, but also on APC function.

Furthermore, total spleen cells harvested 7 days after squalene injection were stimulated in culture with CD3/CD28 Abs. These cultures showed a larger number of proliferating CD4 splenic T-cells positive for intracellular IFN-γ than for IL-4 detected by FACS (**Figure 6B**). This corresponded with a significant 8 fold-increase in T-bet mRNA expression and only a 2 fold-increase in GATA-3 mRNA expression measured by real-time RT-PCR (**Figure S4**).

Together, the results demonstrate that stimulation of CD4 T-cells leads preferentially to a Th1 response when plasma membrane cholesterol is enriched by 40–50%. The Th1 response was more vigorous when APCs were also enriched in membrane cholesterol, as they secreted higher amounts of IL-12, a major cytokine driving Th1 cell differentiation.

### Enrichment of Membrane Cholesterol Fosters the Reactivity of Autoimmune Th1 Cells

To determine whether membrane cholesterol enrichment by *in vivo* squalene administration alters the reactivity of antigen-specific (HA_110–120_) Th1 cells *in vivo*, we took advantage of a RAG2 KO, RIP-HA Tg mouse model in which autoimmune diabetes (type 1 diabetes, T1D) is induced by i.p. infusion of splenic CD4 T-cells (2.5×10^5^ cells) from TCR-HA Tg mice. In this model of inducible T1D, hyperglycemia is detected within 7 to 10 days after transfer of diabetogenic, HA_110–120-_specific Th1 cells [57].

**Figure 7:**
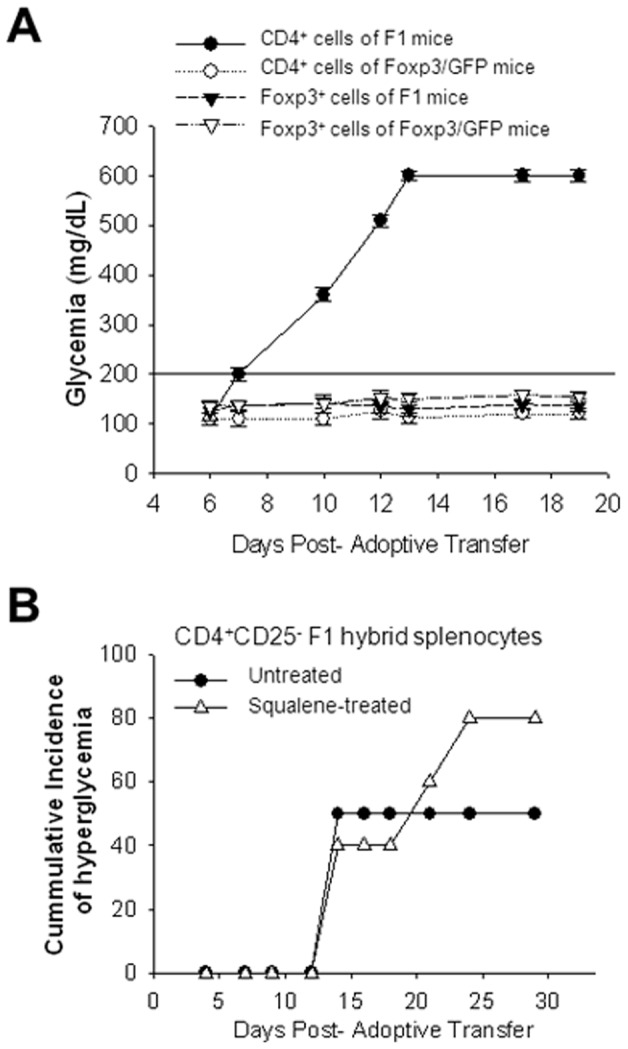
Squalene enrichment of membrane cholesterol fosters the autoreactivity of antigen-specific Th1 diabetogenic cells. (**A**) Diabetogenicity of antigen (HA_110–120_)-specific CD4 splenic Th1 cells was determined in RAG2 KO, RIP-HA mouse model for inducible type 1 diabetes (T1D). The HA_110–120_-specific CD4 splenic T-cells (2×10^5^ cells) from either F1 mice (*Foxp3*-GFP^+/−^ TCR-HA^+/−^ mice) (filled circle), or from parental *Foxp3*-GFP^+^/^+^ mice (open circle) were infused alone, or in combination with *Foxp3*-GFP sorted splenic cells (2×10^5^ cells) from Fl mice (filled triangle) or *Foxp3*-GFP sorted cells (2×10^5^ cells) from parental *Foxp3*-GFP^+^/^+^ mice (open triangle) into RAG2 KO, RIP-HA Tg recipient mice (n = 5 mice/group). Shown is the mean glycemia values for individual mice in each group ± SD. Horizontal dark line indicates the upper limit of euglycemia (200 mg/dL) previously determined in a large cohort of non manipulated RAG2 KO RIP-HA Tg recipients (n = 20). Of note, only the antigen (HA_110–120_)-specific CD4 splenic T-cells, but not T-regs or other non-antigen (HA_110–120_)-specific T-cells were able to induce hyperglycemia in RAG2 KO, RIP-HA Tg recipients. (**B**) Diabetogenicity of antigen (HA_110–120_)-specific CD4 splenic Th1 cells from squalene treated F1 mice was tested in the same RAG2 KO, RIP-HA mouse model for inducible T1D described in panel A. The HA_110–120_-specific CD4 splenic T-cells (2×10^4^ cells) from squalene treated (180 µg/mouse) or untreated F1 mice were first depleted of *Foxp3* ^+^ splenic cells 7 days post-squalene treatment, and then infused i.p. in RAG2 KO, RIP-HA Tg recipients. Shown is the cumulative incidence of hyperglycemia (X-axis) from two separate experiments, n = 5 mice/group/experiment. Cumulative incidence of hyperglycemia was calculated by dividing the number of mice per group that developed two consecutive readings of hyperglycemia (>200 mg/dL) at various intervals of time by the total number of mice and then multiplied by 100.

Using this T1D mouse model, we first tested our F1 hybrid mouse for the expression level and diabetogenicity of TCR-HA^+/−^ CD4^+^ (*Foxp3* ^−/−^) T-cells in the spleen. Similar to the parental TCR-HA^+/+^ homozygous Tg mice, the spleen of F1 hybrid mice expressed ∼40% of TCR-HA^+/−^ CD4^+^ (*Foxp3* ^−/−^) T-cells according to FACS measurements using a TCR-HA clonotypic antibody (# 6.5 mAb). The spleen of F1 hybrid mice also contained a similar number of CD4^+^ *Foxp3*-GFP^+/−^ T-reg cells as the parental *Foxp3*-GFP^+/+^ homozygous Tg mice (1–3%). The diabetogenic potential of TCR-HA^+/−^ Tg CD4 splenic T-cells from F1 hybrid mice (TCR-HA^+/−^, *Foxp3*-GFP^+/−^ dTg mouse) was tested by transferring i.p. 2.5×10^5^ negatively-sorted CD4^+^ (*Foxp3*-GFP)^−/−^ splenic T-cells into RAG2 KO, RIP-HA Tg mice. The RAG2 KO, RIP-HA Tg recipients developed hyperglycemia by 8–10 days after cell transfer, whereas those receiving either CD4^+^ (*Foxp3*-GFP)^ +/−^ T-reg cells from the same F1 donors or CD4 splenic T-reg cells from *Foxp3*-GFP^+/+^ Tg parental mice did not (**Figure 7A**). This clearly demonstrated that only the CD4^+^TCR-HA^+/−^ (*Foxp3-*GFP^−/−^) splenic T-cells, but not the CD4^+^ TCR-HA^+/−^ *Foxp3-*GFP^+/−^ or CD4^+^ *Foxp3-*GFP^+/+^ splenic T-reg cells have diabetogenic potential.

To determine whether enrichment of membrane cholesterol in CD4^+^TCR-HA^+/−^ (*Foxp3-*GFP^−/−^) may affect their diabetogenic potential, splenocytes from untreated and squalene treated F1mice (180 µg squalene/mouse) were isolated 7 days post-injection and depleted of CD4^+^ *Foxp3*-GFP^+/−^ T-regs, followed by negative-sorting of diabetogenic CD4^+^TCR-HA^+/−^ T-cells. Purified diabetogenic CD4^+^(*Foxp3*-depleted) TCR-HA^+/−^ T-cells (1×10^4^ cells/mouse) from squalene treated or untreated F1 mice were then transferred into RAG2 KO, RIP-HA Tg mice, and glycemia (read out for T1D onset) was monitored bi-weekly. Data depicted in **figure 7B** show that the cumulative incidence of hyperglycemia in RAG2 KO, RIP-HA Tg recipients of diabetogenic CD4^+^(*Foxp3*-depleted), TCR-HA^+/−^ T-cells from untreated F1 mice was 50%, whereas in those receiving the same type of cells from squalene treated F1 mice was increased to almost 80%. These results indicated that the enrichment of membrane cholesterol in *self-*reactive (diabetogenic) Th1 cells fostered their diabetogenic potential.

### Cholesterol Enrichment in CD4 Foxp3 T-reg Cell Membrane does not Affect their Suppressogenic Function

Naturally-born CD4 T-cells expressing the master regulatory gene *Foxp3* are the archetype of T-regulatory (T-reg cells). T-reg cells maintain T-cell and B-cell homeostasis and can also restrict immune responses to various *self* and foreign antigens [58]. We have shown above that a single dose of squalene (180 µg/mouse) resulted in a 40 to 50% increase of membrane cholesterol in CD4 *Foxp3* T-reg cells (**Figure 1D**). We next investigated whether their function was altered by membrane cholesterol enrichment. For this, resting CD4 T-cells expressing the HA_110–120_-specific TCR (T effector cells, T-eff) were negatively-sorted from the spleen of untreated or squalene treated (180 µg/mouse) F1 mice 7 days post-injection. FACS-sorted GFP^+^ T-reg cells were also negatively-sorted by FACS from the spleen of untreated or squalene treated (180 µg/mouse) F1 mice 7 days post-injection. The supernatants from 2-day co-cultures of several combinations of T-reg cells and CD4 T-eff cells in the presence of HA_110–120_-pulsed APCs from untreated or squalene treated F1 mice were further assessed for the secretion of IL-2 and major Th1 and Th2 cytokines (IFN-γ and IL-4, respectively) in parallel with mRNA expression level of T-bet, a critical transcription factor that up-regulates IFN-γ synthesis in Th1 cells. We previously reported that the level of T-bet mRNA expression strongly correlates with the suppressogenic capacity of CD4 *Foxp3* T-regs on the proliferation and cytokine secretion of conventional CD4^+^(*Foxp3* ^-^) T-cells (T-eff cells) [48].

**Figure 8:**
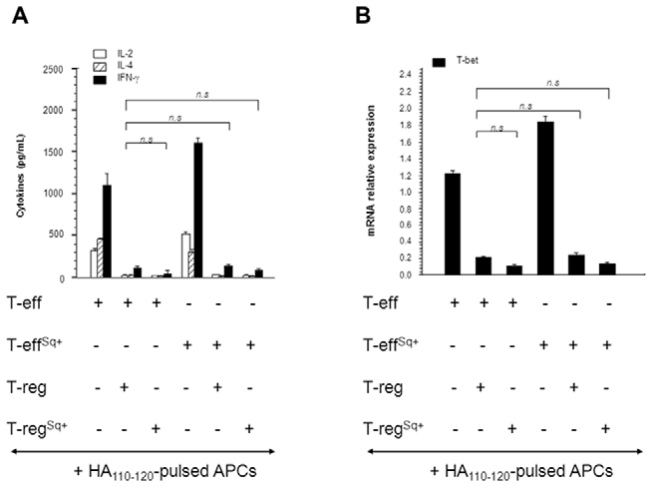
Squalene enrichment of membrane cholesterol in CD4^+^ *Foxp3* ^+^ T-reg cells does not alter their suppressogenic function. (**A**) T-reg suppression of HA_110–120_ -specific CD4 T-effector (T-eff) cells from untreated or squalene treated (single dose of 180 µg/mouse) F1 mice (n = 4/group) has been tested in an *in vitro* suppression assay. Isolated HA_110–120_-pulsed APCs (5×10^5^) were co-cultured for 48 h with FACS-sorted HA-specific CD4^+^ T-eff cells (10^6^ cells) and FACS-sorted *Foxp3*-GFP^+^ T-regs (10^6^ cells) from either untreated or squalene treated (Sq) F1 mice. Various cell co-culture combinations from individual mice are shown in the X-axis, where (+) indicates the presence and (–) indicates the absence of cells in the culture system. Cell-culture supernatants from each Treg/T-eff combination were then measured for secreted IFN-γ (Th1 cytokine) and IL-4 (Th2 cytokine) by Luminex (Y-axis). No significant changes were observed when compared different co-cultures as specified (brackets, *p values>0.05). (**B**) Quantitative real-time RT-PCR measured the T-bet mRNA extracted from triplicate wells of each T-reg/T-eff cells combination described in panel A. The Y axis indicates the mean fold increase in mRNA expression level relative to endogenous 18S rRNA control ± SD. The mRNA relative values were normalized to the untreated T-eff co-cultures with HA-pulsed APCs (reference control sample). Shown is one of two representative experiments. No significant changes were observed when compared different co-cultures as specified (brackets, *p values>0.05).

Interestingly, if considering the effect of membrane cholesterol on CD4 Th1 differentiation in the previous experiments, the CD4 *Foxp3* T-regs were able to induce similar levels of suppression on IL-2, IFN-γ, and IL-4 secretion by conventional CD4 T-cells (T-eff cells) regardless of membrane cholesterol enrichment (**Figure 8A**). This was consistent with a lack of alteration in the T-bet mRNA expression in T-effector cells, as determined by real-time RT-PCR (**Figure 8B**).

## Discussion

Cholesterol-rich microdomains known as lipid rafts are postulated to be a driving force in immunological synapse formation and critical for T-cell signaling [1], [59]–[61].

Squalene is a late precursor of cholesterol that is readily incorporated by hepatocytes and proficiently integrated into the cholesterol biosynthesis pathway after intravenous administration in humans [33]. Herein, we found that a single intraperitoneal injection of squalene leads to a significant enrichment of membrane cholesterol in resting lymphocytes, i.e., CD4 T-cells, CD8 T-cells, B-cells, and dendritic cells in mice. The plasma membrane of CD4 T-cells and CD4 *Foxp3* T-reg cells showed a 40–50% increase in membrane cholesterol above baseline after one dose of squalene, most likely due to an initially lower baseline of membrane cholesterol as compared with other lymphocyte subsets. Squalene enrichment of membrane cholesterol was paralleled by an increased number of resting CD4 splenic T-cells in the absence of T-cell stimulation. This was consistent with early studies showing that provision of cholesterol through high and low density lipoproteins sustains a continuous homeostatic proliferation of T lymphocytes [62]–[64]. Recently, a cholesterol-rich diet or hypercholesterolemia in mice was associated with an increased number of splenic CD4 T-cells [65]. In contrast, atorvastatin-induced inhibition of HMG CoA reductase, a critical enzyme required for cholesterol synthesis, led to a reduction in the number of T-cells in humans [66]. The molecular mechanism(s) by which membrane cholesterol promotes lymphocyte proliferation remains largely unknown. We found that squalene enrichment of membrane cholesterol in resting CD4 T-cells was paralleled by increased IL-2 secretion, a powerful autocrine growth factor for T-cells. This may well explain why the rate of homeostatic proliferation was increased in resting CD4 T-cells enriched in membrane cholesterol.

Spatiotemporal partitioning of protein receptors in lipid rafts influences their signaling status in T-cells, not only in a ligand-specific manner but also by re-partitioning of neighboring receptors and co-receptors [67]. We questioned whether squalene enrichment of membrane cholesterol in the absence of TCR ligation may affect the spatiotemporal partitioning of protein receptors and consequently the tyrosine phosphorylation events critical for recruitment of signaling kinases in CD4 T-cells. The protein synthesis in resting CD4 splenic T-cells was quantitatively unaltered after enrichment of membrane cholesterol. However, fine alterations in tyrosine phosphorylation of several signaling modules associated with receptors involved in Th1/Th2 cell differentiation were detected. Higher amounts of phospho-STAT-4 recruited on IL-12Rβ2, phospho-STAT-5 on IL-2Rα, and phospho-ZAP-70 recruited on TCR/CD3 complex were detected in resting CD4 T-cells enriched in membrane cholesterol, in contrast to phospho-STAT-6 recruited on IL-4Rα that dropped below baseline. Increased ability of IL-12Rβ2 to recruit phospho-STAT-4 and of IL-2Rα to recruit phospho-STAT-5 was consistent with increased IFN-γ, and respectively IL-2 secretion upon CD3/CD28 ligation [68]. These protein receptors have a particular partitioning in the lipid rafts of resting T-cells. Thus, most of TCR α and β chains in resting T-cells reside in the non raft domains, whereas the ζ-chain resides in the raft microdomains [69], [70]. The IL-12Rβ2 is mostly located in the non-raft microdomains, whereas IL-12Rβ1 is located in the raft microdomains, and the ligation through IL-12 leads to a functional IL-12R [71]. The IL-2Rα-chain (CD25) is mostly located in the rafts of resting CD4 T-cells independent of ligation by IL-2 [68], [72], whereas the IL-2Rβ-chain is mostly located in the non-raft microdomains [73]. While the IL-2Rα-chain binds IL-2 with high affinity and lacks signaling ability, the intracellular tail of IL-2Rβ-chain expresses several tyrosine phosphorylated sites as docking sites for JAK kinases, which in turn mediates STAT-5 phosphorylation and dimerization. It has been suggested that a functional IL-2Rαβγ able to promote phosphorylation/dimerization of STAT-5 is assembled mostly in the non-raft microdomains of the plasma membrane [74]. The IL-4Rα chain resides mostly in the rafts and assembles with the common γ chain to form a functional receptor [75].

Interestingly, the ability of the CD28 receptor to recruit PI3K was not affected by membrane cholesterol enrichment in resting CD4 T-cells. The CD28 receptor resides mostly in the raft microdomains during T-cell signaling [76]. These opposing signaling events that occurred solely by cholesterol enrichment in the plasma membrane, strongly suggest that not only receptor ligation but also an increase in rafts cholesterol can lead to spatiotemporal re-partitioning of protein receptors in CD4 T-cells. To this, a large body of evidence indicates that pharmacological alterations in rafts composition like changes in cholesterol content are sufficient to induce re-distribution of protein receptors and their signaling modules in the plasma membrane [reviewed in [77]. A question that remains to be addressed is to what extent squalene enrichment of membrane cholesterol versus squalene itself leads to re-distribution of protein receptors in T-cells? That is because squalene administration in mice up-regulated expression of HMG Co reductase, a critical up-stream enzyme required not only for cholesterol synthesis, but also for generation of small geranylgeranyl and farnesyl radicals actively involved in protein trafficking from cytosol to the plasma membrane [reviewed in [78]. This seems worthy of further investigation, as early studies showed that squalene itself protects rats against cyclophosphamide-induced toxicity by scavenging free radicals and reactive oxidative species [79].

Besides increasing the number of resting CD4 T-cells, squalene enrichment of membrane cholesterol sensitized the Th1 signaling modules in the absence of T-cell stimulation, i.e., STAT-4, STAT-5, and TCR/ZAP-70. Th1 sensitization was significantly augmented in the presence of increased IL-12 secretion by APCs. IL-12 secreted by macrophages and dendritic cells is a well known inflammatory Th1 inducing cytokine whose synthesis is contributed by TLR3 and TLR4 using MyD88 as a common adaptor molecule for down-stream signaling [80]. Although the effect of cholesterol loading in APCs was not the focus of this study, the intimate mechanism by which cholesterol-rich rafts interferes with TLRs signaling and genetic regulation of IL-12 synthesis in APCs is worth being explored. In this study, squalene enrichment of membrane cholesterol in resting CD4 T-cells led to a spatiotemporal re-distribution of IL-2Rβ2 subunit in the rafts according to CLSM analysis, a driving event towards Th1 differentiation. Furthermore, squalene enrichment of membrane cholesterol in the presence of antigen or CD3/CD28 stimulation led to a vigorous Th1 differentiation. This was consistent with an increased reactivity of antigen-specific, diabetogenic Th1 cells in a mouse model for inducible T1D, and with previous findings showing that cholesterol lowering statin therapy can lead to the beneficial reversal of Th1 to Th2 polarization in psoriasis patients associated with hyperlipidemia [81].

*Foxp3* ^+^ T-reg cells represent a particular subset of the CD4 T-cell population. A recent study showed that the number of splenic T-regs peaked at 4 weeks and decreased gradually between 8 and 20 weeks during a cholesterol-rich diet in mice, and this coincided with an increased number of inflammatory Th1 cells over the same period of time [65]. The same study concluded that hypercholesterolemia impairs the T-reg pool but not the migration of inflammatory cells to atherosclerotic lesions. However, our data showed that squalene enrichment of membrane cholesterol did not alter the splenic T-reg frequency. We also found that unlike conventional CD4 T-cells that were polarized toward a Th1 phenotype, the T-reg suppressogenic function remained unaltered after squalene enrichment of membrane cholesterol in CD4 *Foxp3* T-reg cells or CD4 T effector cells.

Together, these data revealed differential regulatory effects of membrane cholesterol on the function of CD4 T-cell subsets, suggesting that membrane cholesterol could be a new therapeutic target to modulate immune functions in various physiopathological conditions.

## Supporting Information

Figure S1 **Squalene administration leads to accumulation of membrane cholesterol in several lymphocyte subsets.** F1 hybrid mice (n = 5/group) were injected i.p. (dark plots) or not (light plots) with a single dose of squalene (180 µg/mouse) and 7 days later splenocytes were individually stained for CD8, CD22.2, CD19, or CD11c, and co-stained with CD3 and Filipin III. Shown are the percent values ± SD and MFI values of Filipin III ± SD collected among 100–800 cell events in gated populations from one of two representative experiments. Values to the left correspond to untreated mice, and values to the right correspond to squalene treated mice.
(TIF)

Figure S2 **Effect of recurrent administration of squalene on the cholesterol metabolism.** **(A)** Serum lipid electrophoresis of F1 mice treated i.p. with 4 doses of squalene (red line) given once a week (180 µg/dose/mouse) (lanes 1–2), or untreated mice (lanes 3–4) as analyzed 7 days after the last squalene injection. Shown are the LDL, VLDL, and HDL serum fractions of cholesterol from 4 of 10 mice analyzed. The electrophoretic bands were scanned by densitometry and quantified using the SCION analysis software (right panel). Of note, a recurrent squalene treatment resulted in increased HDL serum fraction. **(B)** Liver accumulation of cholesterol in individual untreated or squalene treated (180 µg/mouse) F1 mice analyzed 7 days after the last squalene injection by staining frozen liver sections with Sudan IV and hematoxylin (n = 5 mice/group). *Upper* panel shows the presence of cholesterol (reddish spots) in a sample of fat tissue (control cholesterol staining). *Lower left* panel, untreated F1 mouse at X40 magnification. *Lower right* panel, F1 mouse treated with 4 doses of squalene at X40 magnification. Of note, slight increase in cholesterol accumulation (reddish areas) was detected in mice treated with 4 doses. Shown is one representative liver section from each group of mice.
(TIF)

Figure S3 **Squalene enrichment of membrane cholesterol in unstimulated APCs alters their cytokine secretion.** Unstimulated APCs from F1 mice untreated (dark bars) or squalene treated mice (180 µg/mouse) (light bars) were measured for IL-12, IL-6, and IL-1α secretion *in vitro,* 7 days after squalene treatment (n = 5 mice/group). Cell culture supernatants from 2-day cultures of adherent lymphocytes harvested from individual spleens of each group of mice were measured by Luminex. Of note, only IL-12 secretion by unstimulated APCs from squalene treated mice was significantly increased (*p<0.01).
(TIF)

Figure S4 **Alteration in T-bet and GATA-3 mRNA expression levels in splenic cells enriched for membrane cholesterol and stimulated with CD3/CD28 Abs.** Quantitative real-time RT-PCR of T-bet and GATA-3 mRNA extracted from *in vitro* CD3/CD28-stimulated splenocytes from individual F1 mice treated i.p. or not with 1 dose of squalene (180 µg) (n = 5 mice/group) was carried out 7 days after squalene injection. Y axis indicates the mean fold increase in mRNA expression level relative to the endogenous 18S rRNA expression level (control ± SD). Shown are two combined separate experiments (*p value<0.01).
(TIF)

## References

[1] Lingwood D, Simons K (2010). Lipid rafts as a membrane-organizing principle.. Science.

[2] Thomas S, Kumar RS, Brumeanu TD (2004). Role of lipid rafts in T cells.. Arch Immunol Ther Exp (Warsz).

[3] Gidwani A, Holowka D, Baird B (2001). Fluorescence Anisotropy Measurements of Lipid Order in Plasma Membranes and Lipid Rafts from RBL-2H3 Mast Cells†.. Biochemistry.

[4] Prior IA, Muncke C, Parton RG, Hancock JF (2003). Direct visualization of Ras proteins in spatially distinct cell surface microdomains.. J Cell Biol.

[5] Simons K, Toomre D (2000). Lipid rafts and signal transduction.. Nat Rev Mol Cell Biol.

[6] Stevens TJ, Arkin IT (2000). Do more complex organisms have a greater proportion of membrane proteins in their genomes?. Proteins.

[7] Saint-Ruf C, Panigada M, Azogui O, Debey P, von Boehmer H (2000). Different initiation of pre-TCR and gammadeltaTCR signalling.. Nature.

[8] Ebert PJ, Baker JF, Punt JA (2000). Immature CD4+CD8+ thymocytes do not polarize lipid rafts in response to TCR-mediated signals.. J Immunol.

[9] Leitenberg D, Balamuth F, Bottomly K (2001). Changes in the T cell receptor macromolecular signaling complex and membrane microdomains during T cell development and activation.. Semin Immunol.

[10] de Mello Coelho V, Nguyen D, Giri B, Bunbury A, Schaffer E (2004). Quantitative differences in lipid raft components between murine CD4+ and CD8+ T cells.. BMC Immunol.

[11] Poloso NJ, Roche PA (2004). Association of MHC class II-peptide complexes with plasma membrane lipid microdomains.. Curr Opin Immunol.

[12] Gombos I, Detre C, Vamosi G, Matko J (2004). Rafting MHC-II domains in the APC (presynaptic) plasma membrane and the thresholds for T-cell activation and immunological synapse formation.. Immunol Lett.

[13] Anderson HA, Hiltbold EM, Roche PA (2000). Concentration of MHC class II molecules in lipid rafts facilitates antigen presentation.. Nat Immunol.

[14] Horejsi V (2005). Lipid rafts and their roles in T-cell activation.. Microbes Infect.

[15] Mitchell JS, Kanca O, McIntyre BW (2002). Lipid microdomain clustering induces a redistribution of antigen recognition and adhesion molecules on human T lymphocytes.. J Immunol.

[16] Liu SD, Whiting CC, Tomassian T, Pang M, Bissel SJ (2008). Endogenous galectin-1 enforces class I-restricted TCR functional fate decisions in thymocytes.. Blood.

[17] Demetriou M, Granovsky M, Quaggin S, Dennis JW (2001). Negative regulation of T-cell activation and autoimmunity by Mgat5 N-glycosylation.. Nature.

[18] Ho YK, Brown S, Bilheimer DW, Goldstein JL (1976). Regulation of low density lipoprotein receptor activity in freshly isolated human lymphocytes.. J Clin Invest.

[19] Verhoeye FR, Descamps O, Husson B, Hondekijn JC, Ronveaux-Dupal MF (1996). An improved method for detection of low density lipoprotein receptor defects in human T lymphocytes.. J Lipid Res.

[20] Lehoux JG, Kandalaft N, Belisle S, Bellabarba D (1985). Characterization of 3-hydroxy-3-methylglutaryl coenzyme A reductase in human adrenal cortex.. Endocrinology.

[21] Fulop T, Douziech N, Goulet AC, Desgeorges S, Linteau A (2001). Cyclodextrin modulation of T lymphocyte signal transduction with aging.. Mech Ageing Dev.

[22] Nix M, Stoffel W (2000). Perturbation of membrane microdomains reduces mitogenic signaling and increases susceptibility to apoptosis after T cell receptor stimulation.. Cell Death Differ.

[23] Fulop T, Larbi A, Wikby A, Mocchegiani E, Hirokawa K (2005). Dysregulation of T-cell function in the elderly : scientific basis and clinical implications.. Drugs Aging.

[24] Harikumar KG, Puri V, Singh RD, Hanada K, Pagano RE (2005). Differential effects of modification of membrane cholesterol and sphingolipids on the conformation, function, and trafficking of the G protein-coupled cholecystokinin receptor.. J Biol Chem.

[25] Hillyard DZ, Cameron AJ, McDonald KJ, Thomson J, MacIntyre A (2004). Simvastatin inhibits lymphocyte function in normal subjects and patients with cardiovascular disease.. Atherosclerosis.

[26] Jury EC, Isenberg DA, Mauri C, Ehrenstein MR (2006). Atorvastatin restores Lck expression and lipid raft-associated signaling in T cells from patients with systemic lupus erythematosus.. J Immunol.

[27] Kabouridis PS, Janzen J, Magee AL, Ley SC (2000). Cholesterol depletion disrupts lipid rafts and modulates the activity of multiple signaling pathways in T lymphocytes.. Eur J Immunol.

[28] Tani-ichi S, Maruyama K, Kondo N, Nagafuku M, Kabayama K (2005). Structure and function of lipid rafts in human activated T cells.. Int Immunol.

[29] Nguyen DH, Espinoza JC, Taub DD (2004). Cellular cholesterol enrichment impairs T cell activation and chemotaxis.. Mech Ageing Dev.

[30] Van Laethem F, Baus E, Smyth LA, Andris F, Bex F (2001). Glucocorticoids attenuate T cell receptor signaling.. J Exp Med 803–814.

[31] Goldman F, Hohl RJ, Crabtree J, Lewis-Tibesar K, Koretzky G (1996). Lovastatin inhibits T-cell antigen receptor signaling independent of its effects on ras.. Blood.

[32] Brumeanu TD, Goldstein R, Casares S (2006). Down-regulation of autoreactive T-cells by HMG CoA reductase inhibitors.. Clin Immunol.

[33] Eidinoff ML, Knoll JE, Marano BJ, Kvamme E, Rosenfeld RS (1958). Cholesterol biosynthesis: studies related to the metabolic role of squalene.. J Clin Invest.

[34] Mbow ML, De Gregorio E, Valiante NM, Rappuoli R (2010). New adjuvants for human vaccines.. Curr Opin Immunol.

[35] Murakoshi M, Nishino H, Tokuda H, Iwashima A, Okuzumi J (1992). Inhibition by squalene of the tumor-promoting activity of 12-O-tetradecanoylphorbol-13-acetate in mouse-skin carcinogenesis.. Int J Cancer.

[36] Rao CV, Newmark HL, Reddy BS (1998). Chemopreventive effect of squalene on colon cancer.. Carcinogenesis.

[37] Van Duuren BL, Goldschmidt BM (1976). Cocarcinogenic and tumor-promoting agents in tobacco carcinogenesis.. J Natl Cancer Inst.

[38] Loud AV, Bucher NL (1958). The turnover of squalene in relation to the biosynthesis of cholesterol.. J Biol Chem.

[39] Tilvis RS, Miettinen TA (1983). Dietary squalene increases tissue sterols and fecal bile acids in the rat.. Lipids.

[40] Casares S, Hurtado A, McEvoy RC, Sarukhan A, von Boehmer H (2002). Down-regulation of diabetogenic CD4+ T cells by a soluble dimeric peptide-MHC class II chimera.. Nat Immunol.

[41] Kirberg J, Baron A, Jakob S, Rolink A, Karjalainen K (1994). Thymic selection of CD8+ single positive cells with a class II major histocompatibility complex-restricted receptor.. J Exp Med.

[42] Fisson S, Djelti F, Trenado A, Billiard F, Liblau R (2006). Therapeutic potential of self-antigen-specific CD4+ CD25+ regulatory T cells selected in vitro from a polyclonal repertoire.. Eur J Immunol.

[43] Surls J, Nazarov-Stoica C, Kehl M, Casares S, Brumeanu TD (2010). Differential effect of CD4+Foxp3+ T-regulatory cells on the B and T helper cell responses to influenza virus vaccination.. Vaccine.

[44] Casares S, Bona CA, Brumeanu TD (1997). Engineering and characterization of a murine MHC class II-immunoglobulin chimera expressing an immunodominant CD4 T viral epitope.. Protein Eng.

[45] Thomas S, Kumar RS, Casares S, Brumeanu TD (2003). Sensitive detection of GM1 lipid rafts and TCR partitioning in the T cell membrane.. J Immunol Methods.

[46] Castanho MA, Coutinho A, Prieto MJ (1992). Absorption and fluorescence spectra of polyene antibiotics in the presence of cholesterol.. J Biol Chem.

[47] Muller CP, Stephany DA, Winkler DF, Hoeg JM, Demosky SJ (1984). Filipin as a flow microfluorometry probe for cellular cholesterol.. Cytometry.

[48] Nazarov-Stoica C, Surls J, Bona C, Casares S, Brumeanu TD (2009). CD28 signaling in T regulatory precursors requires p56lck and rafts integrity to stabilize the Foxp3 message.. J Immunol.

[49] Brumeanu TD, Preda-Pais A, Stoica C, Bona C, Casares S (2007). Differential partitioning and trafficking of GM gangliosides and cholesterol-rich lipid rafts in thymic and splenic CD4 T cells.. Mol Immunol.

[50] Ness GC, Zhao Z, Keller RK (1994). Effect of squalene synthase inhibition on the expression of hepatic cholesterol biosynthetic enzymes, LDL receptor, and cholesterol 7 alpha hydroxylase.. Arch Biochem Biophys.

[51] Kaplan MH, Sun YL, Hoey T, Grusby MJ (1996). Impaired IL-12 responses and enhanced development of Th2 cells in Stat4-deficient mice.. Nature.

[52] Kaplan MH, Schindler U, Smiley ST, Grusby MJ (1996). Stat6 is required for mediating responses to IL-4 and for development of Th2 cells.. Immunity.

[53] Cantrell DA, Collins MK, Crumpton MJ (1988). Autocrine regulation of T-lymphocyte proliferation: differential induction of IL-2 and IL-2 receptor.. Immunology.

[54] Cantrell D, Smith K (1984). The interleukin-2 T-cell system: a new cell growth model.. Science.

[55] Chan AC, Irving BA, Fraser JD, Weiss A (1991). The zeta chain is associated with a tyrosine kinase and upon T-cell antigen receptor stimulation associates with ZAP-70, a 70-kDa tyrosine phosphoprotein.. Proc Natl Acad Sci U S A.

[56] Lenschow DJ, Walunas TL, Bluestone JA (1996). CD28/B7 system of T cell costimulation.. Annu Rev Immunol.

[57] Bot A, Casares S, Bot S, von Boehmer H, Bona C (1998). Cellular mechanisms involved in protection against influenza virus infection in transgenic mice expressing a TCR receptor specific for class II hemagglutinin peptide in CD4+ and CD8+ T cells.. J Immunol.

[58] Sakaguchi S (2005). Naturally arising Foxp3-expressing CD25+CD4+ regulatory T cells in immunological tolerance to self and non-self.. Nat Immunol.

[59] Zeyda M, Stulnig TM (2006). Lipid Rafts & Co.: an integrated model of membrane organization in T cell activation.. Prog Lipid Res.

[60] Owen DM, Oddos S, Kumar S, Davis DM, Neil MA (2010). High plasma membrane lipid order imaged at the immunological synapse periphery in live T cells.. Mol Membr Biol.

[61] Simons K, Gerl MJ (2010). Revitalizing membrane rafts: new tools and insights.. Nat Rev Mol Cell Biol.

[62] Cuthbert JA, Lipsky PE (1987). Provision of cholesterol to lymphocytes by high density and low density lipoproteins. Requirement for low density lipoprotein receptors.. J Biol Chem.

[63] Cuthbert JA, Lipsky PE (1987). Regulation of lymphocyte proliferation by cholesterol: the role of endogenous sterol metabolism and low density lipoprotein receptors.. Int J Tissue React.

[64] Cuthbert JA, Lipsky PE (1986). Promotion of human T lymphocyte activation and proliferation by fatty acids in low density and high density lipoproteins.. J Biol Chem.

[65] Maganto-Garcia E, Tarrio ML, Grabie N, Bu DX, Lichtman AH (2011). Dynamic changes in regulatory T cells are linked to levels of diet-induced hypercholesterolemia.. Circulation.

[66] Ganesan A, Crum-Cianflone N, Higgins J, Qin J, Rehm C (2011). High dose atorvastatin decreases cellular markers of immune activation without affecting HIV-1 RNA levels: results of a double-blind randomized placebo controlled clinical trial.. J Infect Dis.

[67] Matko J, Szollosi J (2002). Landing of immune receptors and signal proteins on lipid rafts: a safe way to be spatio-temporally coordinated?. Immunol Lett.

[68] Matko J, Bodnar A, Vereb G, Bene L, Vamosi G (2002). GPI-microdomains (membrane rafts) and signaling of the multi-chain interleukin-2 receptor in human lymphoma/leukemia T cell lines.. Eur J Biochem.

[69] Montixi C, Langlet C, Bernard AM, Thimonier J, Dubois C (1998). Engagement of T cell receptor triggers its recruitment to low-density detergent-insoluble membrane domains.. EMBO J.

[70] Xavier R, Brennan T, Li Q, McCormack C, Seed B (1998). Membrane compartmentation is required for efficient T cell activation.. Immunity.

[71] Canda-Sanchez A, Salgado FJ, Perez-Diaz A, Varela-Gonzalez C, Arias P (2009). Differential distribution of both IL-12Rbeta chains in the plasma membrane of human T cells.. J Membr Biol.

[72] Li QR, Ma J, Wang H, Li JS (2005). Interleukin-2alpha receptor in membrane lipid rafts.. Transplant Proc.

[73] Goebel J, Forrest K, Morford L, Roszman TL (2002). Differential localization of IL-2- and -15 receptor chains in membrane rafts of human T cells.. J Leukoc Biol.

[74] Marmor MD, Julius M (2001). Role for lipid rafts in regulating interleukin-2 receptor signaling.. Blood.

[75] Rao R, Logan B, Forrest K, Roszman TL, Goebel J (2004). Lipid rafts in cytokine signaling.. Cytokine Growth Factor Rev.

[76] Viola A, Schroeder S, Sakakibara Y, Lanzavecchia A (1999). T lymphocyte costimulation mediated by reorganization of membrane microdomains.. Science.

[77] Fessler MB, Parks JS (2011). Intracellular lipid flux and membrane microdomains as organizing principles in inflammatory cell signaling.. J Immunol.

[78] Zhang FL, Casey PJ (1996). Protein prenylation: molecular mechanisms and functional consequences.. Annu Rev Biochem.

[79] Senthilkumar S, Devaki T, Manohar BM, Babu MS (2006). Effect of squalene on cyclophosphamide-induced toxicity.. Clin Chim Acta.

[80] Krummen M, Balkow S, Shen L, Heinz S, Loquai C (2010). Release of IL-12 by dendritic cells activated by TLR ligation is dependent on MyD88 signaling, whereas TRIF signaling is indispensable for TLR synergy.. J Leukoc Biol.

[81] Ghazizadeh R, Tosa M, Ghazizadeh M (2011). Clinical improvement in psoriasis with treatment of associated hyperlipidemia.. Am J Med Sci.

