# Supplementary figures and images for: Increased Membrane Cholesterol in Lymphocytes Diverts T-Cells toward an Inflammatory Response
Source: PLoS One. 2012 Jun 19;7(6):e38733. doi: 10.1371/journal.pone.0038733 (PMC3378591; doi:10.1371/journal.pone.0038733)

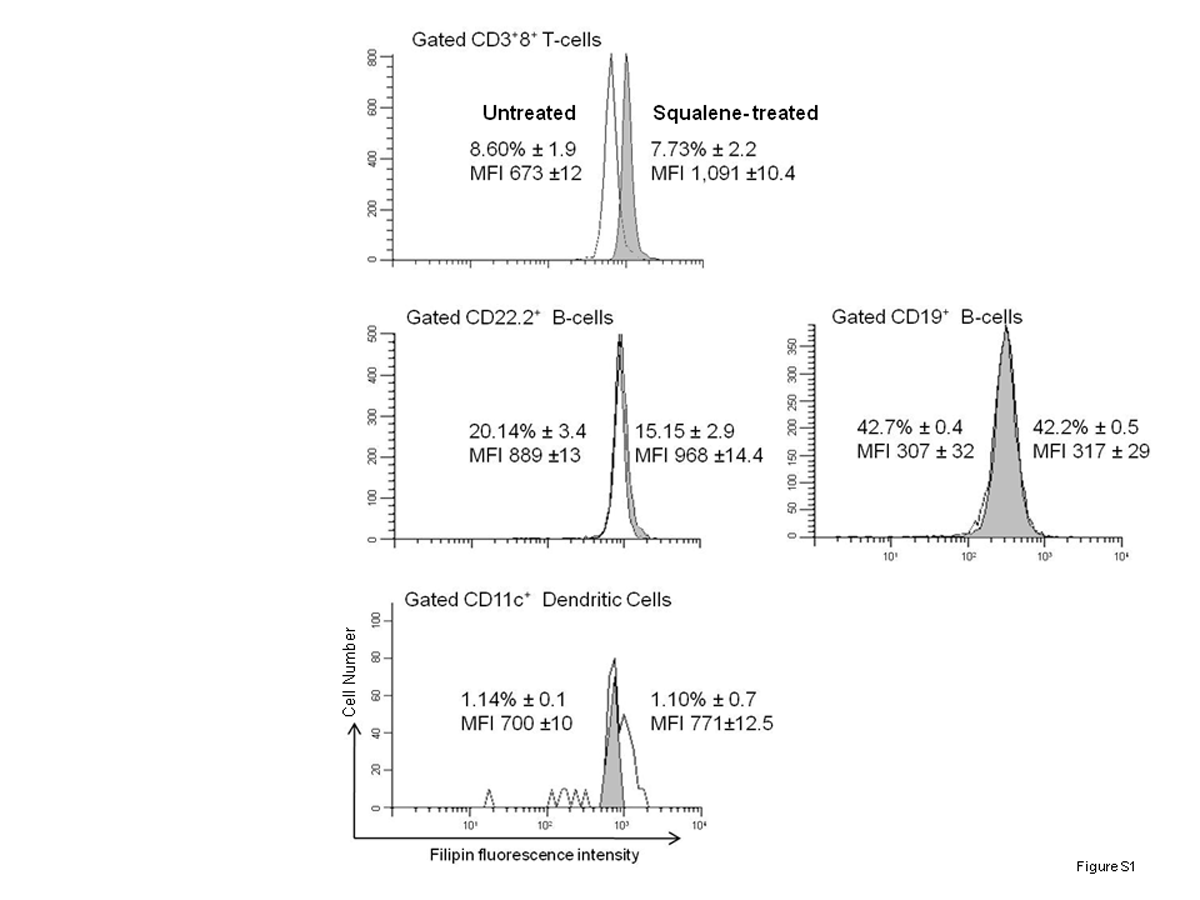

Supplement: Figure S1 — Squalene administration leads to accumulation of membrane cholesterol in several lymphocyte subsets. F1 hybrid mice (n = 5/group) were injected i.p. (dark plots) or not (light plots) with a single dose of squalene (180 µg/mouse) and 7 days later splenocytes were individually stained for CD8, CD22.2, CD19, or CD11c, and co-stained with CD3 and Filipin III. Shown are the percent values ± SD and MFI values of Filipin III ± SD collected among 100–800 cell events in gated populations from one of two representative experiments. Values to the left correspond to untreated mice, and values to the right correspond to squalene treated mice. (TIF) [file pone.0038733.s001.tif]

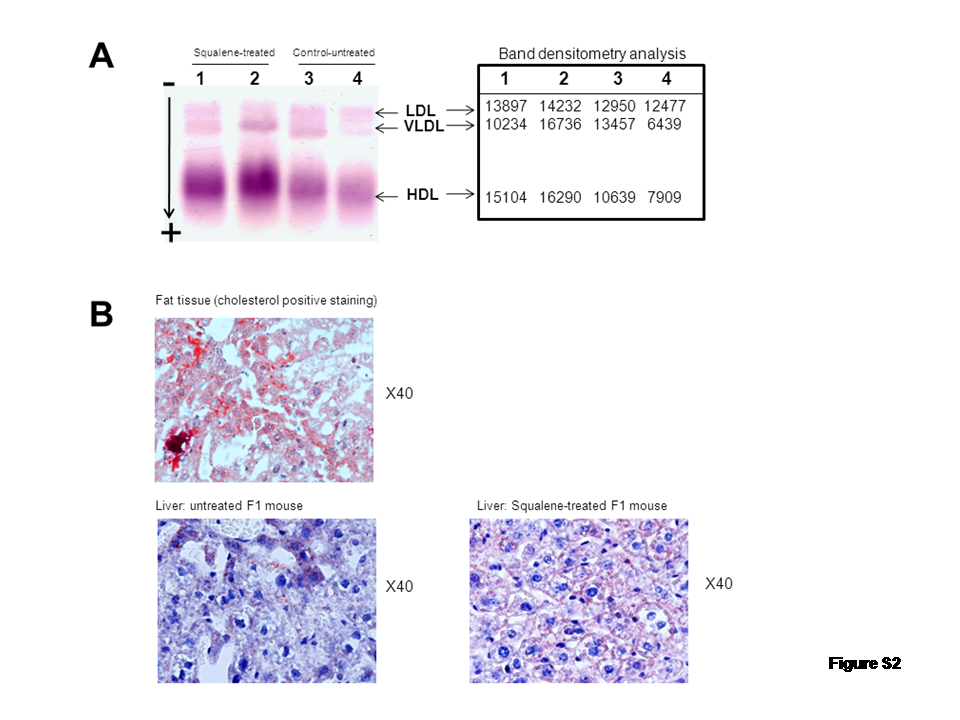

Supplement: Figure S2 — Effect of recurrent administration of squalene on the cholesterol metabolism. (A) Serum lipid electrophoresis of F1 mice treated i.p. with 4 doses of squalene (red line) given once a week (180 µg/dose/mouse) (lanes 1–2), or untreated mice (lanes 3–4) as analyzed 7 days after the last squalene injection. Shown are the LDL, VLDL, and HDL serum fractions of cholesterol from 4 of 10 mice analyzed. The electrophoretic bands were scanned by densitometry and quantified using the SCION analysis software (right panel). Of note, a recurrent squalene treatment resulted in increased HDL serum fraction. (B) Liver accumulation of cholesterol in individual untreated or squalene treated (180 µg/mouse) F1 mice analyzed 7 days after the last squalene injection by staining frozen liver sections with Sudan IV and hematoxylin (n = 5 mice/group). Upper panel shows the presence of cholesterol (reddish spots) in a sample of fat tissue (control cholesterol staining). Lower left panel, untreated F1 mouse at X40 magnification. Lower right panel, F1 mouse treated with 4 doses of squalene at X40 magnification. Of note, slight increase in cholesterol accumulation (reddish areas) was detected in mice treated with 4 doses. Shown is one representative liver section from each group of mice. (TIF) [file pone.0038733.s002.tif]

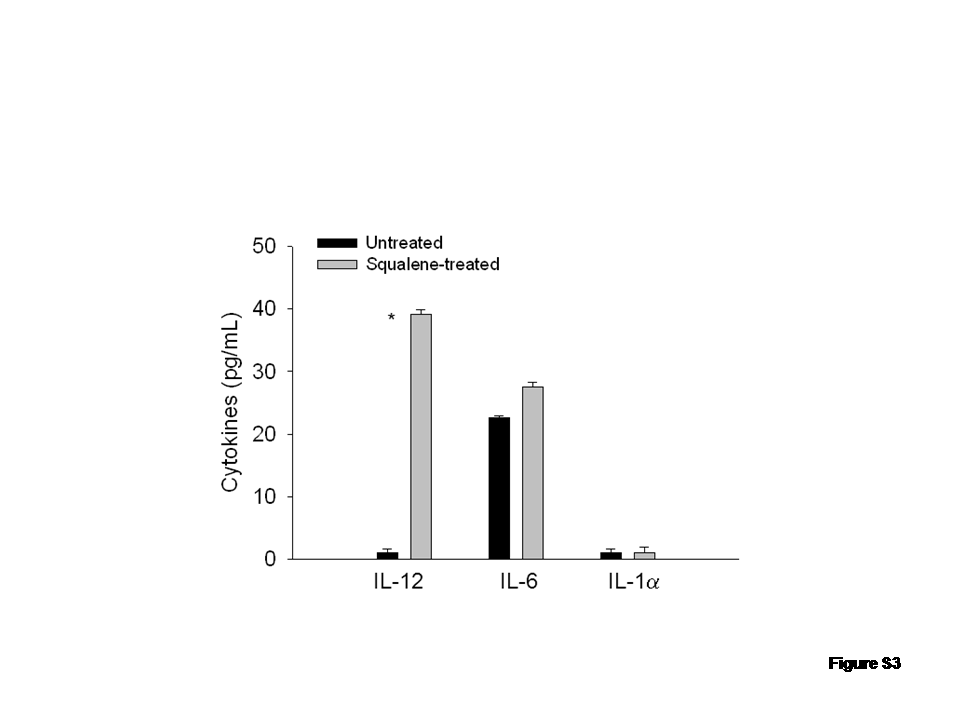

Supplement: Figure S3 — Squalene enrichment of membrane cholesterol in unstimulated APCs alters their cytokine secretion. Unstimulated APCs from F1 mice untreated (dark bars) or squalene treated mice (180 µg/mouse) (light bars) were measured for IL-12, IL-6, and IL-1α secretion in vitro, 7 days after squalene treatment (n = 5 mice/group). Cell culture supernatants from 2-day cultures of adherent lymphocytes harvested from individual spleens of each group of mice were measured by Luminex. Of note, only IL-12 secretion by unstimulated APCs from squalene treated mice was significantly increased (*p<0.01). (TIF) [file pone.0038733.s003.tif]

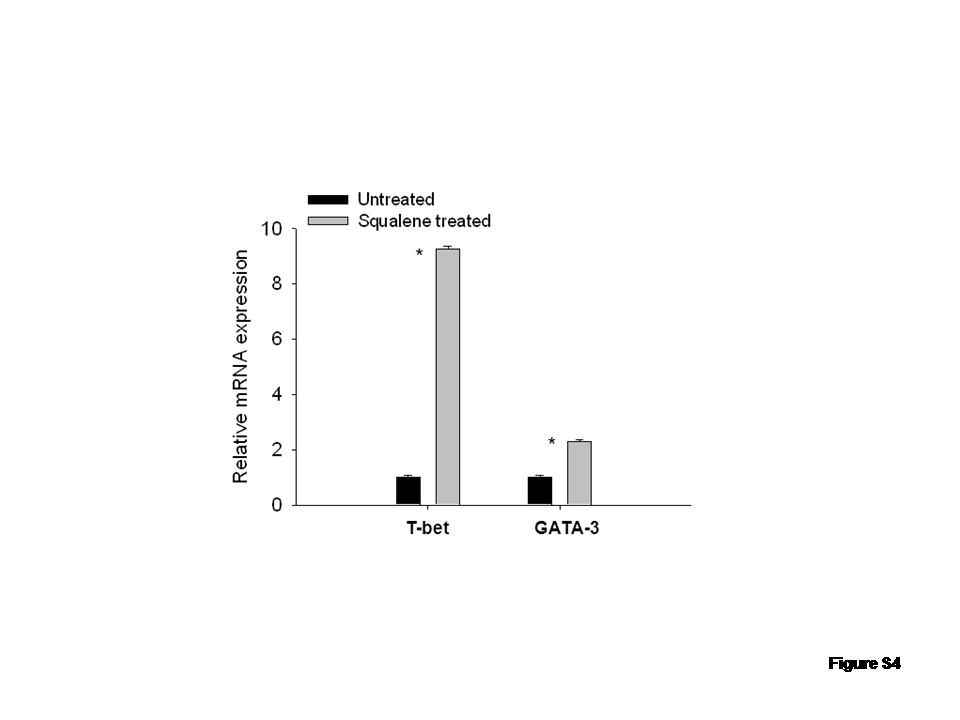

Supplement: Figure S4 — Alteration in T-bet and GATA-3 mRNA expression levels in splenic cells enriched for membrane cholesterol and stimulated with CD3/CD28 Abs. Quantitative real-time RT-PCR of T-bet and GATA-3 mRNA extracted from in vitro CD3/CD28-stimulated splenocytes from individual F1 mice treated i.p. or not with 1 dose of squalene (180 µg) (n = 5 mice/group) was carried out 7 days after squalene injection. Y axis indicates the mean fold increase in mRNA expression level relative to the endogenous 18S rRNA expression level (control ± SD). Shown are two combined separate experiments (*p value<0.01). (TIF) [file pone.0038733.s004.tif]
